# Predicting lymphatic filariasis transmission and elimination dynamics using a multi-model ensemble framework

**DOI:** 10.1016/j.epidem.2017.02.006

**Published:** 2017-03

**Authors:** Morgan E. Smith, Brajendra K. Singh, Michael A. Irvine, Wilma A. Stolk, Swaminathan Subramanian, T. Déirdre Hollingsworth, Edwin Michael

**Affiliations:** a Department of Biological Sciences, University of Notre Dame, Notre Dame, IN 46556, USA; b School of Life Sciences, University of Warwick, Gibbet Hill Road, Coventry CV4 7AL, UK; c Department of Public Health, Erasmus MC, University Medical Center Rotterdam, Rotterdam, The Netherlands; d Vector Control Research Centre (Indian Council of Medical Research), Indira Nagar, Pondicherry 650 006, India; e Mathematics Institute, University of Warwick, Gibbet Hill Road, CV4 7AL Coventry, UK

**Keywords:** Neglected tropical disease, Lymphatic filariasis, Macroparasite dynamics, Multi-model ensemble, Model calibration and validation, Control dynamics

## Abstract

Mathematical models of parasite transmission provide powerful tools for assessing the impacts of interventions. Owing to complexity and uncertainty, no single model may capture all features of transmission and elimination dynamics. Multi-model ensemble modelling offers a framework to help overcome biases of single models. We report on the development of a first multi-model ensemble of three lymphatic filariasis (LF) models (EPIFIL, LYMFASIM, and TRANSFIL), and evaluate its predictive performance in comparison with that of the constituents using calibration and validation data from three case study sites, one each from the three major LF endemic regions: Africa, Southeast Asia and Papua New Guinea (PNG). We assessed the performance of the respective models for predicting the outcomes of annual MDA strategies for various baseline scenarios thought to exemplify the current endemic conditions in the three regions. The results show that the constructed multi-model ensemble outperformed the single models when evaluated across all sites. Single models that best fitted calibration data tended to do less well in simulating the out-of-sample, or validation, intervention data. Scenario modelling results demonstrate that the multi-model ensemble is able to compensate for variance between single models in order to produce more plausible predictions of intervention impacts. Our results highlight the value of an ensemble approach to modelling parasite control dynamics. However, its optimal use will require further methodological improvements as well as consideration of the organizational mechanisms required to ensure that modelling results and data are shared effectively between all stakeholders.

## Highlights

• No single mathematical model captures all features of parasite transmission dynamics.
• Multi-model ensemble modelling can overcome biases of single models.
• A multi-model ensemble of three lymphatic filariasis models is proposed and evaluated.
• The multi-model ensemble outperformed the single models in predicting infection.
• The ensemble approach may improve use of models to inform disease control policy.

## 1 Introduction

There is increasing appreciation that large-scale parasite control or elimination problems belong to a decision and policy domain marked by significant uncertainty, complexity, and spatial heterogeneity (Vespignani, 2012, Klepac et al., 2013, Marathe and Vullikanti, 2013, Bhatt et al., 2015, Heesterbeek et al., 2015). Solving these problems is particularly germane for the current global strategies aiming to eliminate complex vector-borne macroparasitic diseases, such as lymphatic filariasis (LF), which exhibit a high degree of geographic heterogeneity in transmission dynamics and infection patterns, and consequently in extinction dynamics (Gambhir et al., 2010, Irvine et al., 2015, Michael and Singh, 2016, Duerr et al., 2005, Singh and Michael, 2015, Jambulingam et al., 2016, Stolk et al., 2006, Swaminathan et al., 2008). Although mathematical models of transmission can capture many features of these complexities, it is recognized that any single model may be inadequate to fully explore and predict the whole spectrum of system behavior (Oreskes et al., 1994, Neuman, 2003). This is partly a consequence of the inherent complexity of natural systems that give rise to multiple conceptualizations and mathematical descriptions (Oreskes et al., 1994). It is also a reflection of the fact that many different model structures and parameter sets can acceptably reproduce the observed behavior of a complex dynamical system, such that model acceptance in one or more settings may not constitute evidence for general model applicability (Beven and Freer, 2001, Ramin et al., 2012, Hoeting et al., 1999, Christakos, 2003). Indeed, it is increasingly realized in this context that even if approaches based on single models are able to explain the observed behavior of a dynamical system for a given set of data, such models may not generalize well enough to predict future system behavior, particularly under changed conditions – constituting the so-called “out of sample” problem (Simidjievski et al., 2015a, Simidjievski et al., 2015b). Taken together, these uncertainties mean that relying upon forecasts or future predictions generated by a single model for parasite management can lead to significant bias and uncertainty in policy decisions (Lindström et al., 2015).

Recognizing that there may not be a true model of a natural dynamical system, but rather several adequate descriptions reflecting different conceptual bases and structures (Reichert and Omlin, 1997), recent work has focused on using ensemble-based approaches to explicitly account for the uncertainty inherent in the model selection process (Hoeting et al., 1999, Raftery et al., 2005, Gal et al., 2014). Thus, a single-model ensemble involves the use of a number of realizations of an individual model, with distinct predictions obtained for each realization by either introducing stochastic elements, perturbing the input data or initial conditions, or selecting different sets of model parameters (Gal et al., 2014, Viney et al., 2009). By contrast, in a multi-model ensemble, several different models are used, wherein rather than picking the single “best-fitting” model to predict responses, the aim typically is to provide some averaged prediction from different models using various combinatory methods (Hoeting et al., 1999, Raftery et al., 2005). Multi-model ensemble studies, in applications ranging from weather forecasting to cell and population dynamics modelling (Hoeting et al., 1999, Simidjievski et al., 2015a, Simidjievski et al., 2015b, Raftery et al., 2005, Kuepfer et al., 2007), have highlighted the utility of this approach to significantly overcome the problems of over-fitting and model uncertainties, resulting in significant predictive performance gain by these models as compared to that of a single model. Further, studies have shown that even if a multi-model ensemble may not always be the most skillful, its performance is better than the worst single model case, and, as it is often also not possible to predict which of the constituent single-model ensembles will be worst at a given time and location, the use of multi-model ensembles is highly advantageous (Matsueda et al., 2007).

Despite the increasing success of the use of multi-model ensemble methods in other research fields, their application to epidemiological modelling has thus far been limited. However, recent developments in comparing outputs of different influenza models by the MIDAS network (Halloran et al., 2008), assessment of different vaccination strategies (Smith et al., 2012) and impacts of long-term changes in climatic conditions (Ruiz et al., 2014) for malaria, and ensemble-based predictions of Foot and Mouth Disease (FMD) epidemics (Lindström et al., 2015), point to the growing application and value of the method to infectious disease modelling. This body of work demonstrates how combining multiple models can be used to answer critical questions in epidemiology, ranging from the provision of greater confidence in health outcome predictions to improving the ways disease models inform disease control policy, suggesting that the epidemiological use of ensemble-based models are only going to increase in the future.

In this paper, we describe the construction and evaluation of an ensemble of three well-known simulation models of LF epidemiology that incorporate different modelling approaches (deterministic *versus* stochastic), structures (population *versus* individual-based) and parameterization methods (Gambhir et al., 2010, Irvine et al., 2015, Jambulingam et al., 2016, Chan et al., 1998, Norman et al., 2000, Plaisier et al., 1998, Subramanian et al., 2004a, Stolk et al., 2008), in order to better describe the population dynamics of LF and generate more accurate predictions of the impacts of drug and vector-based interventions in communities. The following sections describe the ensemble modelling procedure, analyze prediction accuracy of the single models as well as the multi-model ensemble, and assess the constructed ensemble model’s performance in predicting the population dynamics of LF and the outcomes of various intervention strategies on infection. We end by discussing future work to enhance the ensemble model system for supporting policy-relevant predictions, including potential technical improvements in ensemble construction, and the international coordination mechanisms which will be required to link the system effectively to LF data and to policy making.

## 2 Methods

### 2.1 The models

The three single LF models that make up this study are: EPIFIL, LYMFASIM and TRANSFIL, which are a Monte-Carlo population-based deterministic (EPIFIL), and stochastic individual-based (LYMFASIM, TRANSFIL) models. These models thus differ in complexity from being individual to population-level based, but also in the overall number of parameters used, and in parameterization methods followed. There are also other more subtle differences among the models, including how effects of infection aggregation are handled, and how drug and vector control are incorporated (Gambhir et al., 2010, Irvine et al., 2015, Michael and Singh, 2016, Singh and Michael, 2015, Jambulingam et al., 2016, Stolk et al., 2006, Swaminathan et al., 2008, Norman et al., 2000, Plaisier et al., 1998, Subramanian et al., 2004a, Michael et al., 2004, Singh et al., 2013, Plaisier et al., 2000). These primary inter-model differences are summarized in Table 1, while Table S4 in part B of the Supplementary Information (SI) captures the key similarities and differences in terms of the model parameters used and optimized during model induction and data fitting, and in running simulations of interventions using annual mass drug administrations (MDAs) and vector control. The full details of the three models and their implementation and fitting procedures for LF infection data have been described extensively previously (Gambhir et al., 2010, Irvine et al., 2015, Jambulingam et al., 2016, Swaminathan et al., 2008, Chan et al., 1998, Norman et al., 2000, Plaisier et al., 1998, Subramanian et al., 2004b), and are summarized in part A of the SI.

### 2.2 Experimental setup

**Fig. 1:**
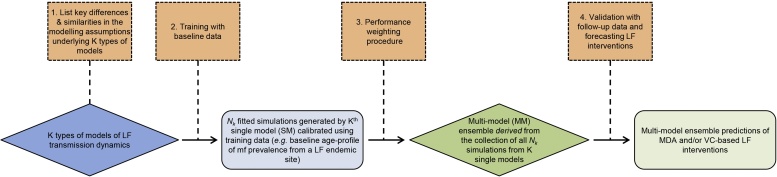
Overview of the methodology used for developing the lymphatic filariasis (LF) multi-model ensemble. All K (=3) single LF models were 1) compared prior to inclusion and 2) trained with baseline data on LF infection (Table 2A) to produce a collection of *N_k_* simulations for each of the three LF model types. Each constituent model was 3) assigned a weight reflecting its relative performance in reproducing the baseline age-mf prevalence data in each site. The weights were used to construct the LF multi-model ensemble. To validate the multi-model ensemble and forecast the effects of LF interventions, 4) simulations were generated by the multi-model ensemble and compared with mf age-prevalence data obtained during the intervention period in each site (Table 2B). The four processes outlined are represented by orange boxes. The different types of models (SM and MM) are represented as blue and green diamonds, respectively, and the corresponding simulation outcomes are represented as rectangles of the same color.

We employed an experimental design in which each LF model was prepared, calibrated and operated by the respective modelling group, following which the relevant simulation outputs from each single model were provided for use in constructing the multi-model LF ensemble. This experimental setup comprised the following steps. First, the three groups were provided with LF baseline infection and post-intervention data from three community sites chosen to represent the vector-mediated transmission dynamics specific to each of the three major LF endemic regions of Africa (primarily *Anopheles*-mediated transmission), Papua New Guinea – PNG – (*Anopheles*) and India (*Culex*) (Singh et al., 2013, Njenga et al., 2008, Rajagopalan et al., 1989, Subramanian et al., 1989, Das et al., 1992, Rajagopalan et al., 1988) (Table 2A and B). The groups were asked to calibrate their models to the baseline microfilariae (mf) age-prevalence data (=“training” data) from these sites, and to provide an ensemble of simulations for the construction and analysis of the multi-model ensemble. Each model aimed to generate 500 fits, or model members, but the number of initial simulations drawn by each group varied from 10,000 (LYMFASIM) to 200,000 (EPIFIL) as a result of differences in the fitting procedures followed and computational intricacies of the three models (see part A of the SI). We deem these as single-model ensembles, which are calibrated and parameterized either using Monte Carlo or stochastic perturbation methods (see parts A and B of the SI). The groups were then asked to use their respective single-model members that fell within the bounds of the weighted multi-model ensemble to carry out simulations of the observed MDA in Malindi, Africa, and Nanaha, PNG, and the effects of the integrated vector management (IVM), which was carried out in Pondicherry, India (Table 2B) (Singh et al., 2013, Njenga et al., 2008, Rajagopalan et al., 1989, Subramanian et al., 1989, Das et al., 1992, Rajagopalan et al., 1988). These simulations were provided for validation against the community mf prevalence data obtained over the durations of the interventions performed in each site (=“validation” data). An overview of the methodology used in this study for calibrating and validating the LF multi-model ensembles constructed for each study site is shown in Fig. 1.

In a second phase of the study, each group was invited to recalibrate their models to a set of overall mf prevalence values thought to represent the currently expected mean infection levels in the regions of Africa, PNG and India, and to provide both their fitted single-model ensembles at baseline and resulting predictions of the effects of MDA in the form of timelines to cross below the WHO-set LF elimination target of 1% mf for each of these scenarios. These single-model ensembles and predictions were then combined using the ensemble construction methodology developed in this study to present an analysis of a multi-model-based generation of these timelines in each region compared to those predicted by each single model.

### 2.3 Study sites and data

We used pre-intervention baseline and post-intervention follow-up mf prevalence data from three study sites, one from each of the three major LF endemic regions of the world (Singh et al., 2013, Njenga et al., 2008, Rajagopalan et al., 1989, Subramanian et al., 1989, Das et al., 1992, Rajagopalan et al., 1988). Note that the infection data used here were age-stratified at baseline and aggregated into overall prevalence for the follow-up years. In addition to infection prevalence information, the baseline annual biting rate (ABR) of vector mosquitoes, along with the vector genus, is known for two of these sites. The baseline survey data (Table 2A) and post-intervention survey data (Table 2B) available for each site served as training and validation data, respectively, for the analysis of the single *versus* multi-model ensemble predictive performances.

### 2.4 Multi-model ensemble construction

Fig. 1 provides a summary of the overall methodological framework followed in the construction of the multi-model LF ensemble in this study. Ensemble construction was essentially performed in two steps:

1. The three LF models were individually calibrated and validated for each given data set, each relying on its own model structure and implementation;
2. The three sets of single-model simulations were then combined using a mean-squared error performance-based weighting scheme as outlined in part C of the SI (Johnson and Swinbank, 2009).

### 2.5 Evaluation metrics

We used three statistical indices to measure the central tendencies of the distributions of errors between data and predictions made by the single-model and multi-model ensembles, and to compare their predictive performance: *viz.* the average error (*AE*), relative error (*RE*), and the relative root mean squared error (*ReRMSE*) (Ramin et al., 2012, Simidjievski et al., 2015a, Simidjievski et al., 2015b, Willmott and Matsuura, 2005). Let *y_i_* be the observed baseline prevalence in age group *i*, *N_k_* be the number of members comprising model *k*, and $x_{kni}^{}$ be the model-predicted prevalence in age group *i* for member *n* of model *k*. The *AE* is calculated as:

$$AE_{k}=\frac{1}{N_{k}I}\sum_{n=1}^{N_{k}}\sum_{i=1}^{I}\left|y_{i}-x_{kni}^{}\right|\text{,}$$

whereas the *RE* is given by:

$$RE_{k}=\frac{1}{N_{k}I}\sum_{n=1}^{N_{k}}\sum_{i=1}^{I}\frac{\left|y_{i}-x_{kni}^{}\right|}{y_{i}}\text{,}$$

and the *ReRMSE* is calculated as:

$$\mathrm{ReRMS}E_{k}=\frac{1}{N_{k}}\sum_{n=1}^{N_{k}}\sqrt{\frac{\sum_{i=1}^{I}{\left(y_{i}-x_{kni}^{}\right)}^{2}}{\sum_{i=1}^{I}{\left(\bar{y}-x_{kni}^{}\right)}^{2}}}\text{,}$$

where $\bar{y}$ represents the overall mean mf prevalence. The *AE* and *RE* measure the average difference (as an absolute and relative measure, respectively) between model predictions and observed values, whereas the *ReRMSE* normalizes these differences by the standard deviation of the system variable (mf prevalence), which thus allows comparisons of model performances either for system variables measured on different scales or for sets of data exhibiting considerable between-study variance in the measured system variable. Smaller values of *ReRMSE* indicate better predictive performance (Simidjievski et al., 2015a, Simidjievski et al., 2015b).

To test if the power of the multi-model ensemble is based on the diversity of the constituent single-model ensembles, we measured the diversity of the single-model ensembles and correlated it to the performance improvement of the multi-model ensemble over the single-model ensembles (Simidjievski et al., 2015a, Simidjievski et al., 2015b). To quantify the diversity of the single models, we measured the average pairwise difference of the single-model members:

$$Diversity=\frac{1}{\left(\frac{N_{k}!}{2!\left(N_{k}-2\right)!}\right)}\sum_{\{m_{1},m_{2}\}\subset N_{k}}\sqrt{\frac{\sum_{n=1}^{I}\left(x_{km_{1}i}^{}-x_{km_{2}i}^{}\right)}{I}}\text{,}$$

where $\left|N_{k}\right|$ denotes the number of members in the single-model ensemble *k*, *I* the number of measurements in the mf age-profile data set, *m*_1_ and *m*_2_ two members in the single-model ensemble *k*, and $x_{km_{1}i}$ and $x_{km_{2}i}^{}$ the simulated values of these outcomes at age-group *i*.

To assess the performance improvement of the multi-model ensemble over a single-model ensemble, we calculate:

$$\mathrm{Improvement}(MM,SM)=-\frac{\mathrm{ReRMS}E_{MM}-\mathrm{ReRMS}E_{SM}}{\mathrm{ReRMS}E_{SM}}$$

where *MM* and *SM*, respectively, represent multi-model and single-model ensembles.

### 2.6 Intervention modelling

In two of the present study sites, Nanaha and Malindi, the LF intervention applied was annual MDA using the drug regimens advocated by WHO for PNG and East Africa respectively, while in Pondicherry, only vector control was implemented through an integrated vector management (IVM) program, which greatly reduced the monthly biting rate of *Culex* mosquitoes during the period (1981–1985) when IVM was operational. Table 1 and part A of the SI text detail the manner by which the effects of these interventions are implemented in the three individual models. For this model comparison exercise, the drug efficacy rates (instantaneous worm and mf kill rates, respectively, denoted by *ω* and *ε*, and the rate of reduction in the fecundity of adult female worms, *δ_reduc_*, for a period of months, *p*) were supplied as fixed values to be used across all modelling groups to ensure that treatment efficacy was similarly applied in all simulations. In Malindi, Kenya, and Nanaha, PNG, the drug-regimens modelled were DEC + ALB and DEC + IVR, respectively. The efficacy values for the two regimens were supplied as follows: DEC + ALB (*ω* = 55%, *ε* = 95%, *δ*_reduc_ = 95%, *p* = 6 months) and DEC + IVR (*ω* = 45%, *ε* = 99%, *δ_reduc_* = 75%, *p* = 9 months) (Michael et al., 2004, Singh et al., 2013, Michael, 2002).

In the case of modelling the impact of the integrated vector management (IVM) interventions carried out during the period 1981–1985 in Pondicherry (Subramanian et al., 1989, Das et al., 1992) on the monthly biting rate (*MBR*), EPIFIL used a simple segmented exponential function of the form, *MBR = MBR*_0_ exp[a_1_t] for each of two periods, to capture the observed decline (for the period, 1981–1985, when the IVM was in effect) and the gradual rise in this variable during the period 1986–1992 when IVM was discontinued (details in part A of the SI). LYMFASIM and TRANSFIL modelled the decline in MBR using the measured reductions in the average MBRs during the period 1981–1985.

### 2.7 Scenario modelling

Predictions of the impacts of annual MDA on timelines (in years) to cross the 1% mf prevalence threshold set by WHO to meet LF elimination were compared between the single- and multi-model ensembles for three hypothetical baseline infection scenarios thought to define current LF conditions in the regions of Africa, Asia and Papua New Guinea. The three scenarios are: (A) an African setting with 10% baseline mf prevalence, where *Anopheles* is the dominant species of LF vector mosquitoes; (B) an Asian setting with 5% baseline mf prevalence, where *Culex* is the dominant species; and (C) a PNG setting with 40% baseline mf prevalence, where *Anopheles* is the dominant species. For all scenarios, the modelled intervention was the application of annual DEC+ ALB with 65% coverage. Note the efficacy rates used for this regimen were the same as those used for the study site of Malindi, Kenya.

## 3 Results

### 3.1 Single-model fits to baseline training data

**Fig. 2:**
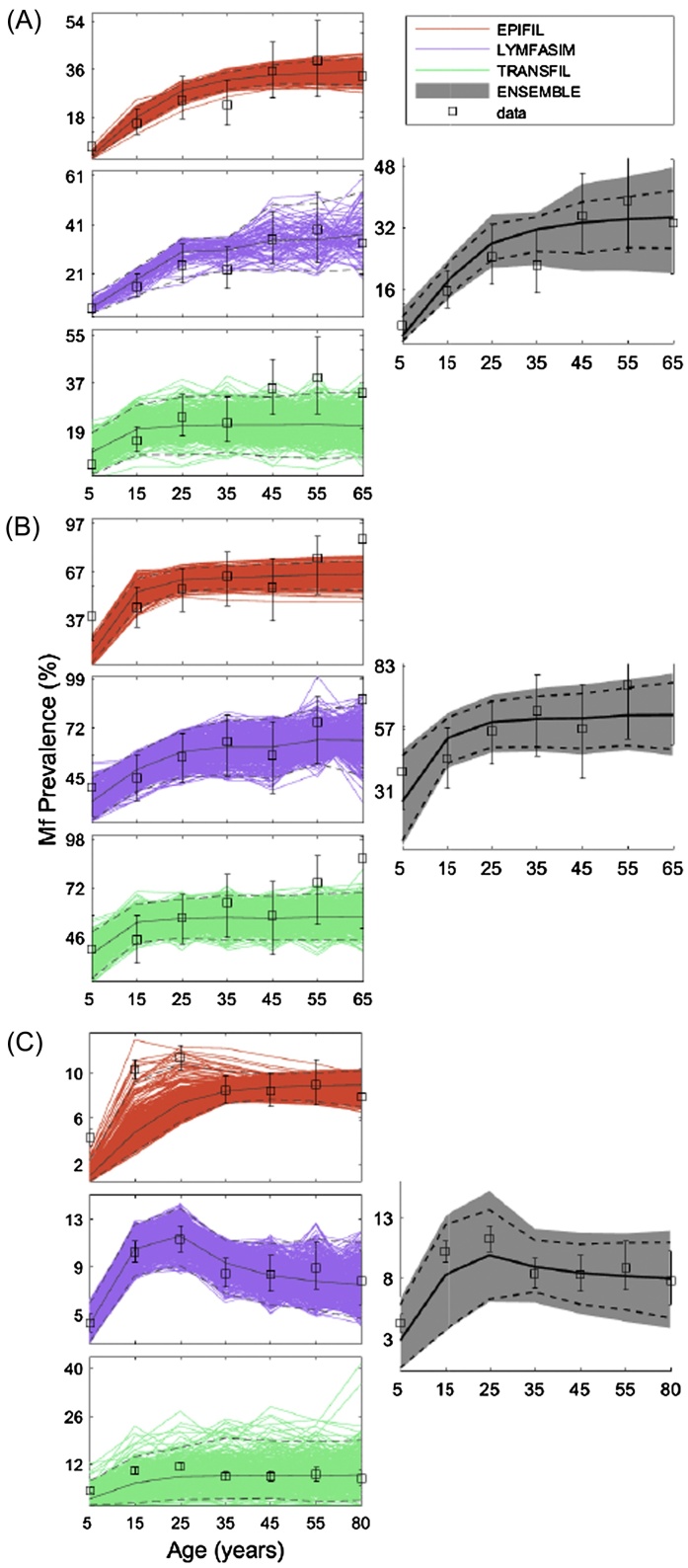
Single-model fits *versus* multi-model ensemble fit to baseline mf age profiles for Malindi, Kenya (A), Nanaha, PNG (B), and Pondicherry, India (C). The observed age-stratified prevalence data are shown as open black squares with 95% confidence interval (CI) bands. The means are depicted by solid black lines, and the 2.5% and 97.5% CIs are portrayed by dashed black lines. Note that there are different limits on the y-axis to better visualize the outputs with 95% CI bands.

The fits of the three single LF transmission models to the baseline mf prevalence data stratified by age in each of the three sites of Malindi, Kenya, Nanaha, PNG, and Pondicherry, India, are shown in Fig. 2. The results show that in the majority of cases the age prevalence data measured in each site fell within the bounds of the single-model ensembles, indicating that each model individually is able to reproduce the infection age patterns observed in these study sites. This is unsurprising given that each group was invited to provide their fitted models for this study, although these varied between 421 and 500 simulations, or members, per model, with the exception of only 82 members from LYMFASIM for the Malindi, Kenya dataset.

The performance statistics calculated for each model displayed in Table 3, however, show that the single models differed in their abilities (based on the *AE*, *RE*, *ReRMSE* metrics) to accurately reproduce the observed data measured in each site. For example, comparing ReRMSE between the models shows that EPIFIL surpasses LYMFASIM and TRANSFIL for two of the sites exhibiting medium (Malindi) to high (Nanaha) infection prevalences, whereas LYMFASIM outperforms the other models for the low prevalence data from Pondicherry. Examination of the diversity index calculated for each model suggests that the predictive performance of the single models for the baseline data may be related inversely to the diversity of the members constituting each single-model ensemble (Table 3). This covariation in model predictive performance and diversity also influenced the weights calculated for each model, with a relatively equal weighting given to each of the three models in the case of Nanaha, whereas the predictions for the Malindi and Pondicherry data were heavily influenced by one model (Table 3). Taken together, these results support the conjecture that it will be difficult to choose a single modelling system for predicting LF infection dynamics that is efficient and robust across all endemic conditions.

To analyze the relationship between the number of members contained in a single-model ensemble and its predictive performance, we used a bootstrap approach to create a range of ensembles for each model with different sizes, *M*. For each *M*, the *ReRMSE* of the resultant member fits to data from the respective sites were calculated and averaged. Table 4 summarizes the results of these experiments, and show that the optimal number of members required for inducing maximal performance varied across models and sites with the smallest set being 50 members for LYMFASIM in Nanaha and the largest set being 350 for LYMFASIM in Malindi and TRANSFIL in Pondicherry. The results indicate, however, that across all three datasets, EPIFIL may achieve optimal performance with 100–200 members, LYMFASIM with 50–350, and TRANSFIL with 150–350.

### 3.2 Multi-model ensemble fits to baseline data

The size of the multi-model ensembles constructed in this study varied between the datasets, comprising a total of 570, 1128, and 888 model members for the Malindi, Nanaha, and Pondicherry sites, respectively. The fits of these multi-model ensembles in comparison to those of the single-model ensembles for the baseline mf age-prevalence data measured in each of the three sites are shown in Fig. 2. The comparative fits visualized in the figure indicate that the multi-model ensembles constructed for each site had, on average, higher performances for predicting these data compared to the single models, with variance also lower than the two most variable single models, LYMFASIM and TRANSFIL. This is formally confirmed by the *ReRMSE* and diversity values obtained by the multi-model ensemble across all three sites compared to the individual models (Table 3), which show that although single models individually surpassed the performance of the multi-model ensemble in a given site, none of these single models achieved this systematically in all instances compared to the multi-model ensemble. The *ReRMSE* values also indicate that for all three datasets, the multi-model ensembles, despite not being the most skillful in each case, always performed better than the corresponding worst single model (Table 3).

### 3.3 Single and multi-model ensemble predictions of site-specific intervention outcomes

**Fig. 3:**
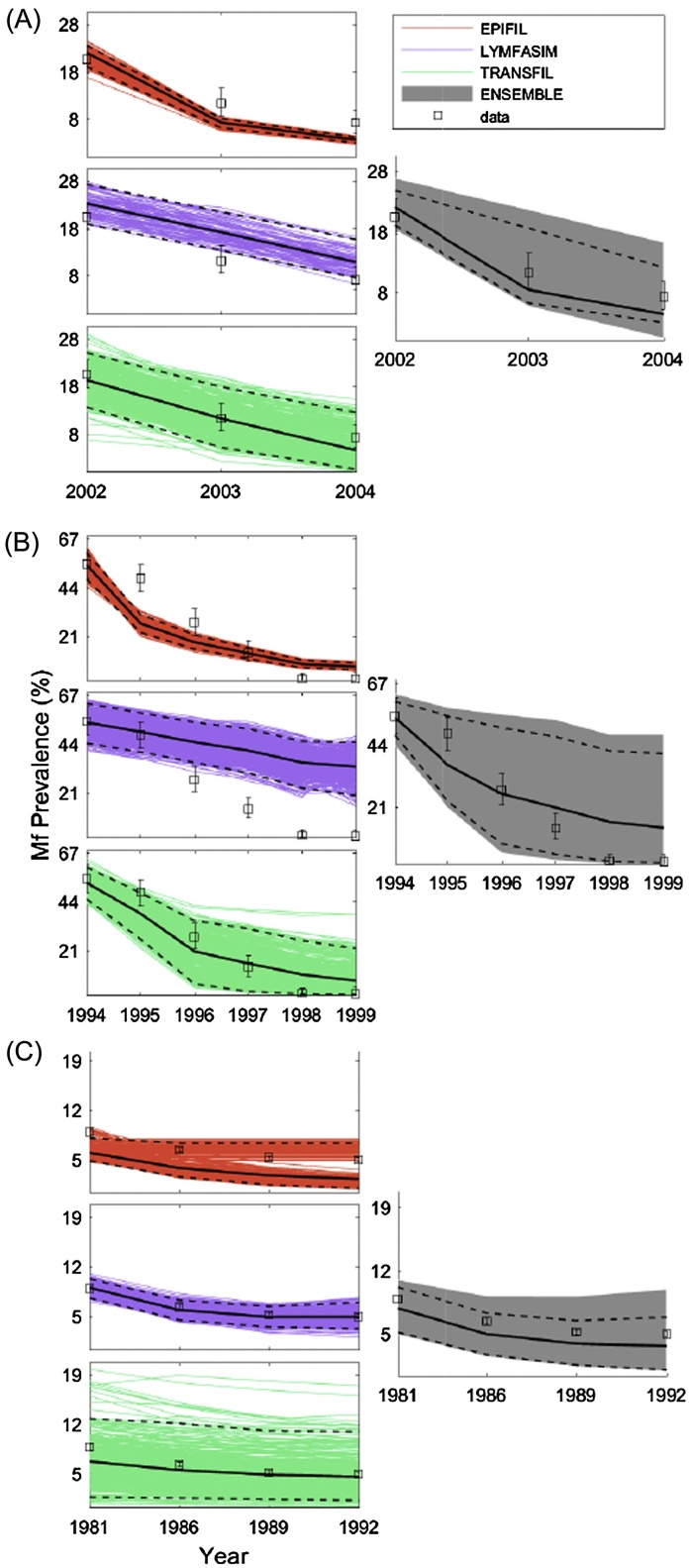
Intervention simulations by the single and multi-model ensembles for Malindi, Kenya (A), Nanaha, PNG (B), and Pondicherry, India (C). The observed overall prevalence data are shown as *open black squares* with 95%-CI bands. The means are shown by *solid black lines*, and the 2.5% and 97.5% CIs are illustrated by *dashed black lines*.

In this exercise, we compared the performances of the single and multi-model ensembles in forecasting the outcomes of the LF interventions applied in each of the three study sites. The effects of MDA based on observed drug coverages were investigated in the case of the Malindi and Nanaha sites (Singh et al., 2013, Njenga et al., 2008), while the impact of IVM was modelled in the case of the Pondicherry site, again using actual implementation coverages and efficacy rates (Rajagopalan et al., 1989, Subramanian et al., 1989, Das et al., 1992, Rajagopalan et al., 1988). Fig. 3 shows the model simulations for these interventions in each site, and confirms our implicit conjecture in this study that using individual models selected on the basis of their performance in fitting training data may lead to inferior prediction performance for data outside the training sample (*i.e.*, exhibiting a significant overfitting problem (Simidjievski et al., 2015a, Simidjievski et al., 2015b). Comparison of the *ReRMSE* values obtained for the single models for the training (baseline) compared to the validation (intervention) data indicate that this might be dependent on the specific modelling system under consideration, with this effect most apparent for EPIFIL.

By contrast, the results show that the multi-model ensembles constructed by combining all three single models are able to compensate for inter-model errors and the effects of any overfitting to training data, and are thus able to predict the effects of interventions, on average, better than the corresponding single models in each site. This is highlighted not only by the results depicted in Fig. 3, which show that the multi-model ensemble is able to envelope all the validation data points from each study site within its bounds (the gray-colored subplot in Fig. 3), but also by the fact that the *ReRMSE* values of the multi-model are never surpassed by the worst performing single model (Table 3). The performance improvement of the multi-model ensemble in comparison to each single model also varied, with LYMFASIM and TRANSFIL outperforming the multi-model ensemble in two of the three sites. EPIFIL, which generally produced the best fits to the baseline training data (Table 3) was surpassed by the multi-model ensemble for all three sites, highlighting that superior model performance on training data can lead to poor predictive performance outside such data.

We next examined if the relative performance of the multi-model ensemble was a function of the varying degree of diversity observed for the single-models in the different study data sets. A negative correlation (correlation coefficient = −0.66) was found between the diversity of the single-model members and the multi-model performance improvement over each of the single-model ensembles across the present data sets in this exercise This negative relationship between the two variables indicates that the multi-model ensemble is able to improve performance over insufficiently diverse single models by exploiting the diversity of the other constituent models (Simidjievski et al., 2015a, Simidjievski et al., 2015b).

### 3.4 Ensemble models and scenario-based predictions of timelines to extinction

The performance statistics for fits of the single and multi-model ensembles to the three hypothetical LF infection scenarios investigated in this study are given in Table 5. The results show that, for the scenario modelling exercise, there was considerable variation between the single models in terms of predictive performances for the regional prevalence data, diversity of good fitting members, and the weights estimated for constructing the LF multi-model ensemble. EPIFIL displayed the best fit, least diversity and highest weighting in direct contrast to TRANSFIL, while LYMFASIM occupied an intermediate position with respect to these metrics (Table 5). As with the site-specific data, the multi-model ensemble again performed better than the averaged skill of the single models for reproducing the hypothetical data, and there was no instance where it was outperformed by the worst single model (in fact, it was the second best performing of the ensembles in the exercise).

**Fig. 4:**
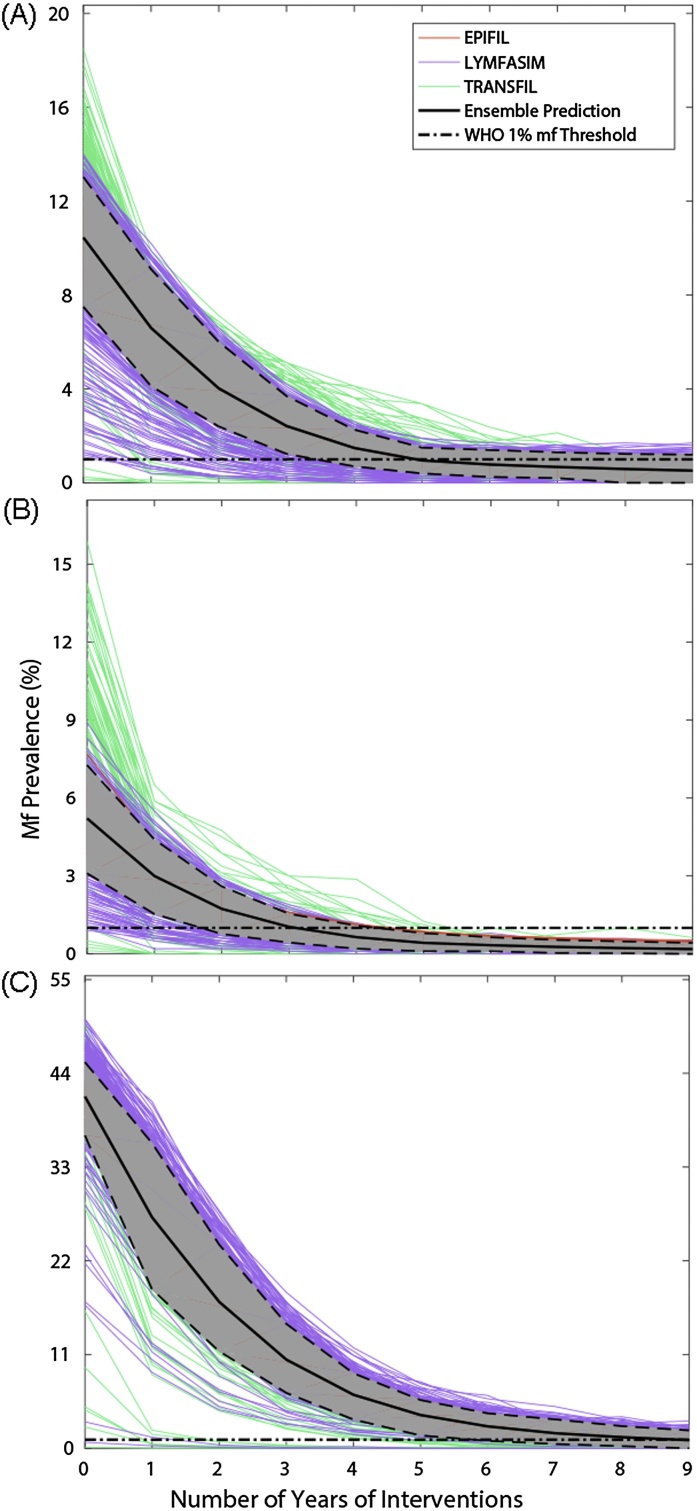
Timelines predicted by the single and multi-model ensemble fits for reaching the WHO threshold of 1% mf prevalence as a result of annual MDA (65% coverage) for three hypothetical settings, one each in sub-Saharan Africa (A), Asia (B), and Papua New Guinea (C). These results are shown for three values of baseline community-level mf prevalence: (A) 10% for a sub-Saharan African setting; (B) 5% for an Asian setting; and (C) 40% for a PNG setting. The horizontal dot-dashed line in each time series graph depicts the WHO 1% mf prevalence threshold.

**Fig. 5:**
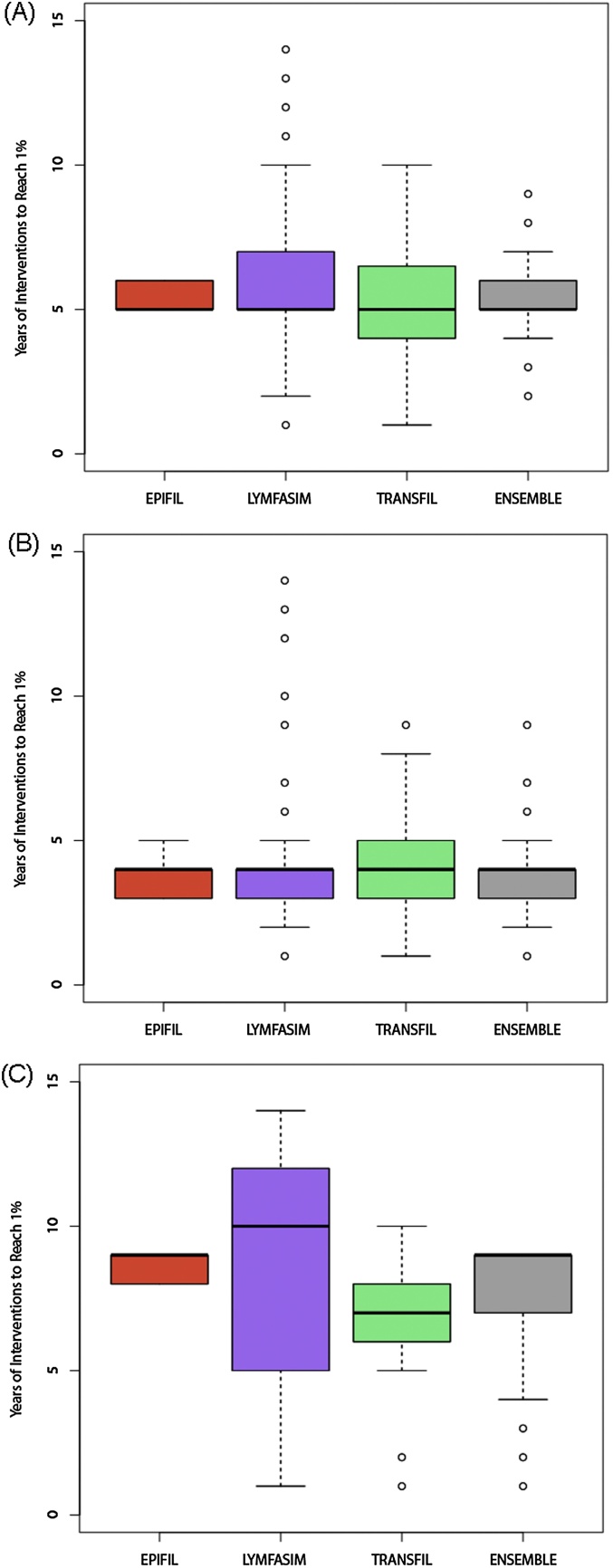
Box plots of median and variance in the number of years of MDA required for crossing below the WHO 1% mf threshold as a result of annual MDA (65% coverage) predicted by the single and multi-model LF ensembles. As in Fig. 4, the required annual rounds are shown for three hypothetical settings, one each in (A) sub-Saharan Africa, (B) Asia, and (C) Papua New Guinea.

Fig. 4 depicts the timelines generated by the single and multi-model ensembles for mf prevalence to cross below the WHO 1% mf threshold, as well as the years required to reach this threshold, for each scenario. Two points are immediately apparent: first, the predictions of single models can vary considerably for each modelled scenario; and second, the corresponding multi-model predictions of the median and variance of the years of MDA required to reach the WHO threshold are dictated by the combined performance and diversity of the single models. Thus, while the best-fitting and least diverse model, EPIFIL, predicts a narrow range of 5–6 years of MDA to reach the 1% mf threshold (from an initial prevalence of 10% mf) in the African scenario, the multi-model ensemble is also informed by the more variable LYMFASIM and TRANSFIL (predictions between 1–10 years of MDA), and concludes a slightly broader range of 4–7 years for this scenario (Figs. 4A and 5 A). In the case of the Asian scenario, the single models predicted anywhere between 1–8 years, whereas the multi-model ensemble combines these predictions to indicate that only 2–5 years of annual MDA will be required (Figs. 4B and 5B). The most dramatic variation in the predictions of the single models were observed for the Papua New Guinea scenario, for which LYMFASIM predicted the need for 1–14 years of MDA to reach the 1% mf threshold, while EPIFIL (and also TRANSFIL to a large degree) predicted a much narrower range of 8–9 years (Figs. 4C and 5C). The multi-model ensemble, by contrast, incorporates these single model uncertainties to predict a range of 4–10 years of MDAs, a result stressing its utility as a device for overcoming single model prediction heterogeneity to produce a combined prediction that takes full account of such errors.

## 4 Discussion

Multi-model ensemble modelling is drawing increasing attention as a mathematical framework for addressing uncertainty when predicting the dynamics of complex systems (IPCC, 2013, Gneiting and Raftery, 2005, Cloke and Pappenberger, 2009, Velazquez et al., 2010). This growing interest also stems from the recognition that when many competing models for a specific complex phenomenon exist, then sampling from the available individual models will help reduce uncertainty or increase the reliability of the modelling results for management decision making (Ramin et al., 2012, Ramin et al., 2011, Velazquez et al., 2011). This is not only because single models when addressed together are likely to represent a wider range of structural configurations and processes leading to a better coverage of the whole behavior of a system, but also because different models will have different strengths and weaknesses and, in an ensemble, the deficiencies in any one model may be balanced by the strengths in others or by compensating errors in another model (Martre et al., 2015). Thus, by reducing the simulation errors of single models, multi-model ensemble modelling is expected to increase the consistency of predictions for a problem and therefore aid in the making of better management decisions.

Here, our major contribution is to develop and implement an initial multi-model ensemble framework for combining simulations of the three currently existing single models of LF transmission in order to produce better predictions of both parasite transmission dynamics and the impacts of interventions aiming to eliminate transmission in different endemic communities and regions. In particular, we describe a combinatory method that allowed calculations of the mean and variance of a LF multi-model ensemble that relied on a weighted combination of simulations from the single models based on their performances for fitting baseline infection prevalence data. The results are then used to evaluate the performance of the constructed multi-model ensemble for providing collective simulations of the effects of interventions compared to each single model. Although we focused on only one combination method, *viz.* construction of a weighted mean ensemble with simulations from constituent single models weighted according to their calibration performance, we show that the performance of the multi-model ensemble for simulating infection prevalence data at both the baseline calibration period and for the intervention validation period was, on average, superior to those of any of the LF single models when evaluations were carried out against field data obtained from endemic communities representative of the three major LF endemic regions of Sub-Saharan Africa, Asia, and Papua New Guinea. Thus, although single-model ensembles outperformed the constructed multi-model ensemble for predicting either the baseline or intervention infection data for a given site-specific dataset, no single model consistently surpassed the collective performance of the multi-model ensemble for reproducing the data across all study sites for each period (Table 3). This result is consistent with findings from previous work on ensemble modelling of complex systems, which has demonstrated how weighted ensemble models often tend, on average, to display significantly higher skill and reliability compared to their constituent single model ensembles, particularly when the single models exhibit considerable disparity in their individual predictions for a dataset or situation (Viney et al., 2009).

Nonetheless, it is apparent that the reliability of the present weighted multi-model ensemble is dependent on both the predictive accuracy as well as variance of the three single LF models. Thus, even though EPIFIL and LYMFASIM contributed most to the ensemble in the case of the Malindi and Pondicherry datasets respectively, the moderate diversity of their fits to the baseline calibration data meant that the variance of the multi-model ensemble predictions for the intervention data in these sites were also relatively lower than the case for Nanaha, where each model not only contributed similarly to the ensemble, but each also exhibited high diversity in their calibration fits leading to a corresponding highly variable ensemble prediction of the validation data (Table 3 and Fig. 3). Although various corrective methods have been proposed to overcome this bias in the contributions of single models to ensemble predictions, our use of the commonly suggested linear regression procedure (Raftery et al., 2005, Vrugt et al., 2008) while decreasing variance also reduced the predictive performance of the constructed LF multi-model ensemble (see part E of the SI), indicating that a more systematic exploration of how best to trim ensemble variability, perhaps *via* exclusion of extreme predictions (Viney et al., 2009) or applying probabilistic approaches (Ramin et al., 2012, Raftery et al., 2005), such as Bayesian model averaging (BMA), will be required to more fully address this problem for improving the forecasting performance of the multi-model ensemble developed in this study. Note, however, that even though we used the intervention data to validate both the single and multi-model ensembles, such an approach may be confounded by the fact that we also asked each modelling group to use fixed drug parameters when simulating the effects of interventions on infection prevalence. If these parameters are incorrect, then the model predictions of trends in infection during the intervention period may reflect the outcomes of this uncertainty rather than the ability to accurately predict the dynamics of control. Future work will need to address this possibility by allowing the single models to estimate these drug parameters directly from intervention data (Singh et al., 2013). Nevertheless, it is clear that the observed improvement of ensemble performance for predicting the validation or intervention data over a single model was related to the diversity of the latter model fits to the calibration, or training, dataset (Table 3). This is in line with the consensus in the ensemble literature that multi-model ensembles perform better by exploiting the diversity of their constituent models (Simidjievski et al., 2015a, Simidjievski et al., 2015b).

Taken together, the above observed interrelationships between diversity and ensemble variance on the one hand, and performance gain on the other, indicates that there may be tradeoffs between these variables. One simple first approach to maximize this tradeoff is to control the number of members contained in the single-model ensemble. Our results gauging the optimal number of members required for producing maximal predictive performance showed that this varied between both the three LF models and the calibration dataset, with EPIFIL needing between 100 and 200 members, LYMFASIM requiring 50–350, and TRANSFIL acquiring between 150 and 350 members to produce best performances across the three sites. Given that each group provided between 421 and 500 good-fitting model members for each site, with the exception of only 82 members in the case of LYMFASIM for the Malindi dataset, this result suggests that the larger variance exhibited by LYMFASIM and TRANSFIL, for Nanaha and Pondicherry in particular, could reflect the combined effects of the different total simulations carried out (which ranged from 10,000 in the case of LYMFASIM up to 200,000 in the case of EPIFIL) in addition to inherent stochastic variation, fewer open parameters, and a larger than required member size used by these models. This indicates that future work will need to pay closer attention to calculating the optimal total simulation and selected member sizes needed by a single model in order to improve the predictive performances of both the single and the resultant multi-model ensembles.

The multi-model ensemble analysis carried out in this study also produced new insights regarding the potential predictive performances of the three constituent LF models. In particular, the results showed that EPIFIL may have the tendency to overfit a given training dataset leading to lower diversity in the ensemble members and therefore a lower capacity for reproducing ‘out-of-sample’ intervention predictions (Fig. 2, Fig. 3, and Table 3). By contrast, LYMFASIM and TRANSFIL, while fitting the training data generally less well, had sufficiently diverse members to generate better fitting predictions of the intervention/validation data (Fig. 2, Fig. 3, and Table 3). Since these LF models differed only slightly in structure, with only the ways in which the aggregation parameter and drug coverage are implemented differing significantly between the models (part A of the SI), the observed differential abilities of the models for simulating the training and intervention data must therefore reflect the different means by which the models are fitted to data. Specifically, this may be related to the fact that the Bayesian Melding method by which EPIFIL is fitted to data facilitates generation of a larger number of optimizable parameters (Gambhir et al., 2010, Michael and Singh, 2016, Singh and Michael, 2015, Singh et al., 2013) as compared to distributed models, such as LYMFASIM and TRANSFIL, which have many parameters that must be prescribed *a priori* and only few optimizable parameters to compensate for the computationally intensive nature of individual-based models (see part A of the SI). These implementation differences mean that EPIFIL is better able to fit a given calibration dataset, and, given the Importance Sampling algorithm employed to select the best-fitting members, is also able to obtain fits with low between-member diversity. In contrast, with most parameters fixed, LYMFASIM and TRANSFIL were able to fit the training dataset only by selection of a more diverse set of compensatory members. These results have the implication that there will be a need to increase the diversity of members of EPIFIL while commensurately the fitted members of LYMFASIM and TRANSFIL will require to be trimmed to improve their predictive performances if these single models are to be used individually or separately for making predictions of the effects of LF interventions.

The ability of multi-model ensembles as an effective means to overcome these biases of single models, however, is amply demonstrated by the regional scenario modelling results obtained in this study (Fig. 4). The key finding here is that although the predictions of the multi-model ensemble is influenced greatly by the accuracy of the best-fitting single model for reproducing calibration or training data, the variance in the ensemble predictions is also informed by the more variable model members (Table 5 and Fig. 4). Thus, while the best calibrated model for each scenario prevalence, EPIFIL, predicted a much lower range in the number of annual MDA cycles required to reach below the 1% mf threshold set by WHO as a global target for determining LF transmission elimination in all three of the regional scenarios investigated here, the multi-model ensemble borrowing from the contributions of the more variable LYMFASIM and TRANSFIL concluded slightly broader ranges in each case, intermediate between the predictions of EPIFIL *versus* LYMFASIM and TRANSFIL. These results show that a weighted multi-model ensemble can overcome the low diversity of a model like EPIFIL, while at the same time is also able to trim the variance of distributed models like LYMFASIM and TRANSFIL, to allow a synthesis of predictions that take account of both current uncertainties and biases pertaining to the conceptualization and implementation of available single models as well as a likely better coverage of the whole system space (Martre et al., 2015).

Given that the scenario modelling work carried out in this study is effectively an inverse solution exercise, it is important to assess the respective predictions for consistency and coherence in order to determine the reliability of the results for supporting parasite management. Here, we highlight a major finding in this regard consistent with previous work in modelling LF interventions, *viz.* that following the single model predictions the multi-model ensemble forecasts of the number of annual MDAs required to meet the WHO 1% mf target across the three regional scenarios also tended to be positively related to baseline prevalence (Irvine et al., 2015, Michael and Singh, 2016, Singh and Michael, 2015, Jambulingam et al., 2016, Michael et al., 2004, Singh et al., 2013). Thus, while the multi-model ensemble predicts between 2–5 years for the Asian scenario (for an initial mf prevalence of 5%), it predicted between 4–7 years and 4–10 years of MDAs required for meeting the WHO target in the case of the African (initial prevalence of 10%) and PNG (initial prevalence of 40%). Although these are first findings, and as the respective single and multi-model predictions take explicit account of differences in vector genus composition between the three scenarios investigated, these results indicate, as previously demonstrated by the single models, that timelines to set targets will be greatly influenced by baseline endemicity. This implies strongly that any variations in baseline prevalences in a setting will lead to site-specific differences in the durations of annual MDA required to break LF transmission (Michael and Singh, 2016, Singh and Michael, 2015, Michael et al., 2004). The policy implication of this result is clear, *viz.* the WHO recommended six years of MDA is highly unlikely to break transmission of LF similarly everywhere, and that a more flexible approach that takes full account of spatial heterogeneities in baseline initial conditions will be required to affect the global elimination of LF.

In conclusion, this study has shown how, by reducing simulation error and improving the accuracy and consistency of simulation results, multi-model ensembles based on a deterministic weighted combination of single model predictions may offer a powerful tool for reproducing LF infection patterns in different settings and for forecasting the effects of interventions efficiently. This work represents our first attempt to develop a multi-model LF ensemble, and it is clear that future research will be required to extend the weighting scheme and the performance standard used to improve the initial framework presented here. In particular, we indicate that this work will benefit by adopting the emerging statistical post-processing methods developed in the field of ensemble weather forecasting, including incorporating various bias correction methods and methods from Bayesian calibration and ensemble modelling, to yield a more robust approach for the synthesis of multiple parasitic transmission models (Ramin et al., 2012, Lindström et al., 2015, Raftery et al., 2005, Viney et al., 2009, Martre et al., 2015, Vrugt et al., 2008). Since the accuracy of multi-model ensembles depends on the accuracy of the constituent single models, future research should also focus on better calibration of LF models for generating the ensemble of model outputs (Makowski, 2015). A continued effort to develop reliable single models will increase their individual ability to support reliable decision making in parasite control and, in turn, improve the skill of multi-model ensembles to do so as well. While these technical improvements will enhance reliable LF ensemble modelling, it is also important to consider the organizational and collaborative mechanisms required to develop such ensemble frameworks so that predictions may be delivered to policy makers effectively. A first need in this regard is perhaps the provision of an open forum for exchange of observations, including LF monitoring data from endemic countries, models, and ensemble configuration and results to the entire LF community. Currently, sharing of such observations and model results is done primarily on a bi-lateral basis through individual contact, although modelling collaboratives, such as the Gates Foundation funded Neglected Tropical Disease Modelling Consortium, is clearly beginning to overcome this issue. Recent developments based on Science Gateways (Manset et al., 2012, Foster, 2005, McLennan and Kennell, 2010), by which collaborative tools are made available *via* the internet, however, may provide a more effective way to achieve this, and should be explored in earnest if ensemble-based modelling is to become an effective tool for guiding decisions in the global fight against parasitic diseases.

## **Authors**’ contributions

All authors conceived the study. EM, BKS, and MES designed the analysis design and methodology. MES, BKS, MAI, SS, WAS, and EM performed the model simulations and analysis. EM, BKS, and MES drafted the manuscript. All authors reviewed and approved the final manuscript.

## Conflicting interests

None.

## Figures and Tables

**Table 1:** Models studied and their characteristic features.

Model	Infection dynamics in humans	Infection dynamics in vectors	Implementation	Total number of parameters (number fitted)^a^	Intervention (MDA or VC)	Refs
EPIFIL	Deterministic, age-structured Partial differential equations (PDE)	Deterministic Ordinary differential equation (ODE)	A Monte Carlo-based Bayesian Melding framework using a binomial likelihood function to fit data	28 (24)	Random MDA coverage, with reduction in the biting rate as observed due to VC^b^ where applicable	Gambhir et al. (2010), Chan et al. (1998) and Norman et al. (2000)

LYMFASIM	Stochastic, individual-based micro-simulation	Deterministic non-linear	A chi-squared statistic based fitting method	19 (3)	MDA compliance is neither completely random nor completely systematic, with reduction in the biting rate as observed due to VC^c^ where applicable	Jambulingam et al. (2016), Plaisier et al. (1998) and Subramanian et al. (2004b)

TRANSFIL	Individual-based micro-simulation	Deterministic ODE	An Approximate Bayesian Computation based fitting procedure	14 (3)	Systematic non-compliance of MDA, with reduction in the biting rate as observed due to VC^c^ where applicable	Irvine et al. (2015)

a Those parameters that are not fitted to data have fixed values.

b In EPIFIL, the impact of IVM in Pondicherry was modelled using the equation: *MBR_VC_ = MBR*_0_ exp[a_1_t], with a_1_ < 0 for ∀*t* when VC is ON, otherwise a_1_ > 0. Details can be found in part A of the SI text describing EPIFIL.

c In both LYMFASIM and TRANSFIL, IVM in Pondicherry was modelled as the observed reduction in the average MBRs during the period 1981–1985.

**Table 2:** A. Baseline survey data for the three study sites. B. Follow-up survey data for three study sites.

A. Baseline survey data for the three study sites						
Site	Malindi, Kenya		Nanaha, PNG		Pondicherry, India	
Vector Genus	Anopheles		Anopheles		Culex	
ABR	–		11611		26400	
Baseline Year	2002		1994		1981	
Age Group	Sample Size	Mf Prevalence (%)	Sample Size	Mf Prevalence (%)	Sample Size	Mf Prevalence (%)
<10	135	6.7	28	39.3	2651	4.3
10–20	225	15.6	56	44.6	4560	10.2
20–30	115	24.3	50	56.0	3422	11.3
30–40	94	22.3	28	64.3	2033	8.4
40–50	77	35.1	21	57.1	1364	8.4
50–60	41	39.0	20	75.0	806	8.9
60+	36	33.3	8	87.5	589	7.8
Overall	723	20.5	211	55.0	15425	8.9

B. Follow-up survey data for three study sites.												
Site	Malindi, Kenya				Nanaha, PNG				Pondicherry, India			
Intervention	DEC + ALB^a^ MDA				DEC + IVR^a^ MDA				Integrated vector management			
	Follow-Up Year	Sample Size	Mf Prevalence (%)	MDA coverage (%)	Follow-Up Year	Sample Size	Mf Prevalence (%)	MDA coverage (%)	Follow-Up Year	Sample Size	Mf Prevalence (%)	IVM coverage (%)
	2003	461	11.3	83.4	1995	238	48.3	71.6	1986	22464	6.4	0^b^
	2004	484	7.2	80.3	1996	208	27.4	65.8	1989	20416	5.6	0^b^
					1997	196	13.3	62.8	1992	15885	5.0	0^b^
					1998	221	0.9	66.8				
					1999	166	0.6	49.4				

a Diethylcarbamazine (DEC), albendazole (ALB), ivermectin (IVR).

b Integrated Vector Management (IVM) was active in Pondicherry 1981–1985. Declines in mf prevalence were observed in the years following.

**Table 3:** Performance statistics of single and multi-model ensemble predictions with respect to the baseline (training) data and post-intervention (validation) data.

Site	Model	Weight^a^	*Diversity*	*AE*	*RE*	*ReRMSE*	*Improvement* (MM > SM)
Baseline performance							
Malindi	EPIFIL	0.60	2.34	3.98	0.16	0.45	−0.07
	LYMFASIM	0.29	7.46	5.58	0.22	0.60	0.21
	TRANSFIL	0.11	7.03	8.97	0.36	1.43	0.67
	MM Ensemble	–	3.08	4.15	0.17	0.48	–

Nanaha	EPIFIL	0.32	5.25	11.69	0.19	0.81	−0.28
	LYMFASIM	0.41	10.10	9.71	0.16	0.90	−0.15
	TRANSFIL	0.27	8.23	11.70	0.19	1.43	0.28
	MM Ensemble	–	8.91	10.83	0.18	1.04	–

Pondicherry	EPIFIL	0.17	1.14	2.26	0.27	0.88	0.22
	LYMFASIM	0.75	1.86	1.20	0.14	0.56	−0.23
	TRANSFIL	0.08	5.14	3.61	0.43	0.91	0.25
	MM Ensemble	–	2.41	1.61	0.19	0.68	–

Post-intervention performance							
Malindi	EPIFIL	–	0.50	3.78	0.41	0.90	0.01
	LYMFASIM	–	2.47	4.81	0.52	0.83	−0.07
	TRANSFIL	–	3.93	3.25	0.35	0.71	−0.26
	MM Ensemble	–	2.10	3.87	0.42	0.89	–

Nanaha	EPIFIL	–	1.83	8.93	0.49	1.33	0.20
	LYMFASIM	–	7.01	23.23	1.28	1.10	0.03
	TRANSFIL	–	8.53	8.04	0.44	0.72	−0.48
	MM Ensemble	–	13.64	12.24	0.68	1.07	–

Pondicherry	EPIFIL	–	1.56	2.93	0.53	1.13	0.18
	LYMFASIM	–	0.96	0.70	0.13	0.90	−0.02
	TRANSFIL	–	2.70	1.97	0.36	0.95	0.03
	MM Ensemble	–	1.89	1.66	0.30	0.93	–

a Weights derived during calibration are used in validation.

**Table 4:** Predictive performance of the single models given varying numbers of members. The *ReRMSE* values in bold face indicate the best performing set of single-model members at the given site.

Site	No. model members	EPIFIL *ReRMSE*	LYMFASIM *ReRMSE*	TRANSFIL *ReRMSE*
Malindi	50	0.439	0.584	1.031
	100	0.450	0.591	1.038
	150	0.433	0.587	1.012
	200	0.445	0.582	1.216
	250	0.447	0.613	1.118
	300	0.447	0.586	**1.001**
	350	0.444	**0.575**	1.164
	400	0.439	0.590	1.072
	450	0.452	0.598	1.140
	500	0.446	0.579	1.105

Nanaha	50	0.810	**0.877**	1.360
	100	**0.784**	0.905	1.579
	150	0.824	0.966	**1.359**
	200	0.806	0.966	1.417
	250	0.803	0.967	1.404
	300	0.812	0.946	1.462
	350	0.803	0.948	1.381
	400	0.805	0.939	1.396
	450	0.807	0.930	1.429
	500	0.806	0.979	1.432

Pondicherry	50	0.846	0.558	0.861
	100	0.859	**0.532**	0.808
	150	0.857	0.566	0.733
	200	**0.835**	0.541	0.748
	250	0.838	0.557	0.760
	300	0.856	0.559	0.727
	350	0.859	0.551	**0.723**
	400	0.876	0.566	0.797
	450	0.848	0.557	0.814
	500	0.860	0.564	0.751

**Table 5:** Performance statistics of the single and multi-model fits to hypothetical LF scenario data.

Scenario (% mf prevalence)	Model	Weight	*Diversity*	*AE*	*RE*
Africa (10%)	EPIFIL	0.833	1.191	0.896	0.090
	LYMFASIM	0.130	2.865	1.984	0.198
	TRANSFIL	0.037	4.517	4.485	0.449
	MM Ensemble	–	1.627	1.229	0.123

Asia (5%)	EPIFIL	0.748	0.975	0.746	0.149
	LYMFASIM	0.231	1.723	1.196	0.239
	TRANSFIL	0.020	3.998	4.672	0.934
	MM Ensemble	–	1.179	0.866	0.173

PNG (40%)	EPIFIL	0.914	1.903	1.438	0.036
	LYMFASIM	0.060	5.203	4.914	0.123
	TRANSFIL	0.026	8.225	6.358	0.159
	MM Ensemble	–	2.874	2.367	0.059
