# Supplementary material for: Predicting lymphatic filariasis transmission and elimination dynamics using a multi-model ensemble framework
Source: Epidemics. 2017 Mar;18:16–28. doi: 10.1016/j.epidem.2017.02.006 (PMC5340857; doi:10.1016/j.epidem.2017.02.006)
Supplement: Supplementary file 1 [file mmc1.pdf]

Supplementary Information

A. Model descriptions

EPIFIL model description

The mathematical model of LF transmission dynamics

We employed a genus specific mosquito-vectored transmission model of LF to carry out the modeling work in this study [1-5]. Briefly, the state variables of this hybrid coupled partial differential and differential equation model vary over age (a) and/or time (t), representing changes in the pre-patent worm burden per human host $(P(a,t))$, adult worm burden per human host $(W(a,t))$, the microfilariae (Mf) level in the human host modified to reflect infection detection in a 1ml blood sample $(M(a,t))$, the average number of infective L3 larval stages per mosquito (L), and a measure of immunity $(I(a,t))$ developed by human hosts against L3 larvae. The state equations comprising this model are:

$$\frac{\partial P}{\partial t}+\frac{\partial P}{\partial a}=\lambda\frac{V}{H}h(a)\Omega(a,t)-\mu P(a,t)-\lambda\frac{V}{H}h(a)\Omega(a,t-\tau)\zeta$$
$$\frac{\partial W}{\partial t}+\frac{\partial W}{\partial a}=\lambda\frac{V}{H}h(a)\Omega(a,t-\tau)\zeta-\mu W(a,t)$$
$$\frac{\partial M}{\partial t}+\frac{\partial M}{\partial a}=\alpha s\phi[W(a,t),k]W(a,t)-\gamma M(a,t)$$
$$\frac{\partial I}{\partial t}+\frac{\partial I}{\partial a}=W_T(a,t)-\delta I(a,t)$$
$$\frac{dL}{dt}=\lambda\kappa g\int\pi(a)(1-f[M(a,t)])da-(\sigma+\lambda\psi_1)L$$
$$L^*=\frac{\lambda\kappa g\int\pi(a)(1-f[M(a,t)])da}{\sigma+\lambda\psi_1}$$

The above equations involve partial derivatives of four state variables (P - pre-patent worm load; W - adult worm load; M - microfilaria intensity; I - immunity to acquiring new infection due to the pre-existing total worm load where $W_T = W(a,t) + P(a,t)$). Given the faster time scale of infection dynamics in the vector compared to the human host, the infective L3-stage larval density in mosquito population is modeled by an ordinary differential equation essentially reflecting the significantly faster time-scale of

the infection dynamics in the vector hosts. This allows us to make the simplifying assumption that the density of infective stage larvae in the vector population reaches a dynamic equilibrium (denoted by L^*) rapidly [1,2,5-7]. This basic coupled immigration-death structure of the model as well as its recent extensions has been extensively discussed previously [1,2,4-7]. The effects of worm patency are captured by considering that at any time *t*, human individuals of age less than or equal to the pre-patency period, τ, will have no adult worms or Mf, and the rate at which pre-patent worms survive to become adult worms in these individuals at $a > \tau$ is given by $\zeta = \exp(-\mu\tau)$. The term $f(M)$ enables us to account for the different establishment and development rates of the incoming L3-stage larvae as adult worms depending on the genus of mosquito vectors as expressed below:

$$f[M(a,t)] = \left[\frac{2}{\left[1 + \frac{M(a,t)}{k}\left(1 - \exp\left[-\frac{r}{\kappa}\right]\right)\right]^k} - \frac{1}{\left[1 + \frac{M(a,t)}{k}\left(1 - \exp\left[-\frac{2r}{\kappa}\right]\right)\right]^k} \right]$$ for mosquitoes of *Anopheline* genus;

$$f[M(a,t)] = \left(1 + \frac{M(a,t)}{k}\left(1 - \exp\left[-\frac{r}{\kappa}\right]\right)\right)^{-k}$$ for mosquitoes of *Culicine* genus.

In the above, $k[= k_0 + k_{Lin} M]$ is the shape parameter of the negative binomial distribution on the Mf uptake whereas r and κ are respectively the rate of initial increase and the maximum level of L3 larvae. See **Table S1** for the description of all the model parameters and functions. The details of the derivation of the two uptake functions are given elsewhere [2].

Bayesian Melding fitting of the LF model to the baseline data

We follow the BM procedure of model fitting to data, outlined in detail in our previous work [1,5]. In brief, we begin by using the known ranges of the parameter values to generate distributions of parameter priors. We then randomly sample with replacement from these prior distributions to generate 200,000 parameter

vectors, which are then run using the ABR values, if given, for a site to generate model outputs. The model outputs are then melded with age-stratified Mf prevalence data, by calculating binomial log-likelihoods for each parameter vector. In the resampling step of the BM method, a Sampling-Importance-Resampling algorithm is then used to perform 500 draws with replacement to select from among the pool of parameter vectors generated as above, with probabilities proportional to their relative log likelihood values. This step generates the most likely parameter vectors describing the data. These resampled parameter vectors are then used to generate distributions of variables of interest from the fitted model (eg. age-prevalence curves, worm breakpoints and infection trajectories following treatments).

Modeling intervention by mass drug administration

Intervention by mass drug administration was modeled based on the assumptions that anti-filarial treatment with a combination drug regimen acts by killing certain fractions of the populations of adult worms and microfilariae instantly after the drug administration. These effects are incorporated into the basic model by calculating the population sizes of worms and microfilariae as follows:

$$\left.\begin{aligned} P(a,t+dt) &= (1-\omega C)P(a,t) \\ W(a,t+dt) &= (1-\omega C)W(a,t) \\ M(a,t+dt) &= (1-\varepsilon C)M(a,t) \end{aligned}\right\} \quad \text{at time } t = T_{MDA_i}$$

where dt is a short time-period since the time-point T_{MDA_i} when the ith MDA was administered. During this short time-interval, a given proportion of adult worms and microfilariae are instantly removed. The parameters ω and ε, are drug killing efficacy rates for the two life stages of the parasite while the parameter C represents the MDA coverage. Apart from instantaneous killing of microfilariae, the drug continues to kill the newly reproduced Mf by any surviving adult worms at a rate δ_{reduc} for a period of time, p. We model this effect as follows:

$$\frac{\partial M(a,t)}{\partial t} + \frac{\partial M(a,t)}{\partial a} = (1-\delta_{reduc}C)s\alpha\phi(W(a,t),k)W(a,t) - \gamma M(a,t)\,, \quad \text{for } T_{MDA_i} < t \le T_{MDA_i} + p$$

We simulate LF intervention by running the model with the fixed-values of the four drug-related parameters (ω, ε, δ_{reduc}, and p) for MDA coverage levels given by data. The first MDA round is implemented into the model by affecting the population sizes of worm and microfilariae from the baseline fits, and then the intervention is simulated forward in time for a number of years, with subsequent MDA rounds implemented annually.

Modeling vector control

To implement simulations of the impact of the integrated vector management (IVM) interventions carried out during the period 1981 - 1985 in Pondicherry[8,9] on the monthly biting rate (*MBR*) in EPFIL, we fitted a segmented exponential function (*i.e.* $MBR = MBR_0 \exp[a_1 t]$), capturing the observed decline (for the period 1981 to 1985, when the IVM was in effect, with measured MBR_0 in 1980 (= 2200) [8,9] and $a_1 = -2.1$), and then the gradual rise in observed *MBR* for the period 1986 to 1992 when IVM was discontinued, with MBR_0 in 1989 (= 345)[8,9] and $a_1 = 0.12$.

Table S1 - Description the basic LF model parameters and functions used in the model.

Parameter	Definition (*units*)	Range	Refs
λ	Number of bites per mosquito (*per month*)	[5, 15]	1,2,5,10,11
τ	Pre-patency period	[6, 9]	12
s	Proportion of female worms	0.5	-
μ	The worm mortality rate (*per month*)	[0.008, 0.018]	1,2,5,13-16
α	Production rate of microfilariae per worm (*per month*)	[0.25, 1.5]	1,2,5,17
γ	The death rate of the microfilariae (*per month*)	[0.08, 0.12]	1,5,15,17
g	Proportion of mosquitoes which pick up infection when biting an infected host	[0.259, 0.481]	1,5,18
κ	Maximum level of L3 given Mf density	[3.955, 4.83]	1,5
k_0	The basic location parameter of negative binomial distribution used in aggregation parameter ($k = k_0 + k_{Lin} M$)	[0.000036, 0.00077]	1,5,8,9
δ	Immunity waning rate (*per month*)	[0, 0.000001]	1,5
V/H	Ratio of number of vector to hosts	$MBR^{\#} / \lambda$	data
k_{Lin}	The linear rate of increase in the aggregation parameter defined above	[0.00000024, 0.282]	1,5,8,9
σ	Death rate of mosquitoes (*per month*)	[1.5, 8.5]	1,5,19
ψ_1	Proportion of L3 leaving mosquito per bite	[0.12, 0.7]	17
ψ_2	The establishment rate[1]	[0.0000398, 0.00364]	1,2,5,19
H_{Lin}	A threshold value used in $h(a)$ to adjust the rate at which individuals of age *a* are bitten: linear rise from	[12, 240] months	1,5,7

	0 at age zero to 1 at age H_{lin} in years. $h(a) = a / H_{Lin}$ for $a < H_{Lin}; h(a) = 1$ for $a \geq H_{Lin}$		
r	Gradient of Mf uptake[2]	[0.0495, 0.22]	[1,5]
c	Strength of acquired immunity	[0.0000003, 0.0109]	[1,5]
$\boldsymbol{I_C}$	Strength of immunosuppression[3]	[0.5, 5.5]	[1,5]
$\boldsymbol{S_C}$	Slope of immunosuppression function[4] (*per worm/month*)	[0.01, 0.19]	[1,5]
MDA drug-related parameters			
$\boldsymbol{\omega}$	Worm killing efficacy (instantaneous)	dependent on drug regimen	[4]
$\boldsymbol{\varepsilon}$	Microfilariae killing efficacy (instantaneous)	dependent on drug regimen	[4]
$\boldsymbol{\delta_{reduc}}$	Reduction in the worm's fecundity over a period of time p	dependent on drug regimen	[4]
p	A time period during which the drug remains efficacious in reducing the fecundity of the surviving adult worms	dependent on drug regimen	[4]
C	Percentage of the population administered the drug	data	data
**Implementing vector control (VC)** such as IVM modifies the V/H ($= MBR/\lambda$)			
MBR_{VC}	$MBR_{VC} = MBR_0 \exp[a_1 t]$, with $a_1 < 0$ for $\forall t$ when VC is ON, otherwise $a_1 > 0$.	data and estimates	[8,9]
**Description**	**Mathematical expressions of the functions**	**Parameters**	
Probability that an individual is of age *a* $\pi(a)$	$\pi(a) = A_0 \exp[-B_0 a]$	Human age *a* in month, A_0 and B_0 estimated from country demographic data	[1,5,7]
Larvae establishment rate (modified by acquired immunity) Ω(a,t)	$L^* \psi_1 \psi_2 g_1(I) g_2(W_T)$	ψ_1 - proportion of L3 leaving mosquito per bite; ψ_2 - the establishment rate[1]	
Adult worm mating probability $\phi(W,k)$	$1 - \left(1 + \frac{W}{2k}\right)^{-(1+k)}$	*k* – negative binomial aggregation parameter	[1,2,5,20]
Immunity to larval establishment $g_1(I)$	$\frac{1}{1 + cI}$	*c* – strength of immunity to larval establishment	[1,5]
Host immunosuppression $g_2(W_T)$	$\frac{1 + I_C S_C W_T}{1 + S_C W_T}$	I_C – strength of immunosuppression; S_C – slope of immunosuppression	[1,5]

[1]The proportion of L3-stage larvae infecting human hosts that survive to develop into adult worms[1].
[2]The gradient of Mf uptake *r* is a measure of the initial increase in the infective L3 larvae uptake by vector as *M* increases from 0[1,7].
[3] The facilitated establishment rate of adult worms due to parasite-induced immunosuppression in a heavily infected human host
[4] The initial rate of increase by which the strength of immunosuppression is achieved as *W* increases from 0[21].
Note MBR (monthly biting rate) serves as an input to initialize the model, measured as mosquito bites per person per month, the value of which may be obtained from entomological surveys conducted in study sites. In the absence of the observed MBR value, the model has been adapted to estimate it from the community-level Mf prevalence data.

Literature Cited

1. Gambhir M, Bockarie M, Tisch D, Kazura J, Remais J, Spear R, Michael E. (2010) Geographic and ecologic heterogeneity in elimination thresholds for the major vector-borne helminthic disease, lymphatic filariasis. BMC Biology 8: 22.

2. Gambhir M, Michael E. (2008) Complex ecological dynamics and eradicability of the vector borne macroparasitic disease, lymphatic filariasis. PLoS One 3(8): e2874.

3. Michael E, Malecela-Lazaro MN, Kabali C, Snow LC, Kazura JW. (2006) Mathematical models and lymphatic filariasis control: Endpoints and optimal interventions. Trends in Parasitology 22(5): 226-233.

4. Michael E, Malecela-Lazaro MN, Simonsen PE, Pedersen EM, Barker G, Kumar A, Kazura JW. (2004) Mathematical modelling and the control of lymphatic filariasis. Lancet Infectious Diseases 4(4): 223-34.

5. Singh BK, Bockarie MJ, Gambhir M, Siba PM, Tisch DJ, Kazura J, Michael E. (2013) Sequential modeling of the effects of mass drug treatments on anopheline-mediated lymphatic filariasis infection in papua new guinea. PLoS One 8(6): e67004.

6. Chan MS, Srividya A, Norman RA, Pani SP, Ramaiah KD, Vanamail P, Michael E, Das PK, Bundy DA. (1998) Epifil: A dynamic model of infection and disease in lymphatic filariasis. The American Journal of Tropical Medicine and Hygiene 59(4): 606-14.

7. Norman RA, Chan MS, Srividya A, Pani SP, Ramaiah KD, Vanamail P, Michael E, Das PK, Bundy DA. (2000) EPIFIL: The development of an age-structured model for describing the transmission dynamics and control of lymphatic filariasis. Epidemiology & Infection 124(3): 529-41.

8. Subramanian S, Pani SP, Das PK, Rajagopalan PK. (1989) Bancroftian filariasis in pondicherry, south india: 2. epidemiological evaluation of the effect of vector control. Epidemiology and Infection 103(3): 693-702.

9. Das PK, Manoharan A, Subramanian S, Ramaiah KD, Pani SP, Rajavel AR, Rajagopalan PK. (1992) Bancroftian filariasis in pondicherry, south india: Epidemiological impact of recovery of the vector population. Epidemiology and Infection 108(3): 483-493.

10. Rajagopalan PK. (1980) Population dynamics of culex pipiens fatigans, the filariasis vector, in pondicherry - influence of climate and environment. Proceedings of the Indian National Science Academy B46: 745-752.

11. Subramanian S, Manoharan A, Ramaiah KD, Das PK. (1994) Rates of acquisition and loss of wuchereria bancrofti infection in culex quinquefasciatus. The American Journal of Tropical Medicine and Hygiene 51(2): 244-249.

12. Scott AL. (2000) Lymphatic-dwelling filariae. In: Nutman TB, editor. Lymphatic Filariasis. London: Imperial College Press. pp. 5-39.

13. Vanamail P, Subramanian S, Das PK, Pani SP, Rajagopalan PK. (1990) Estimation of fecundic life span of wuchereria bancrofti from longitudinal study of human infection in an endemic area of pondicherry (south india). Indian Journal of Medical Research 91(July): 293-297.

14. Evans DB, Gelband H, Vlassoff C. (1993) Social and economic factors and the control of lymphatic filariasis: A review. Acta Tropica 53(1): 1-26.

15. Ottesen EA, Ramachandran CP. (1995) Lymphatic filariasis infection and disease - control strategies. Parasitology Today 11(4): 129-131.

16. Vanamail P, Ramaiah KD, Pani SP, Das PK, Grenfell BT, Bundy DAP. (1996) Estimation of the fecund life span of wuchereria bancrofti in an endemic area. Transactions of the Royal Society of Tropical Medicine and Hygiene 90(2): 119-121.

17. Hairston NG, de Meillon B. (1968) On the inefficiency of transmission of wuchereria bancrofti from mosquito to human host. Bulletin of the World Health Organization 38(6): 935-941.

18. Subramanian S, Krishnamoorthy K, Ramaiah KD, Habbema JDF, Das PK, Plaisier AP. (1998) The relationship between microfilarial load in the human host and uptake and development of *wuchereria bancrofti* microfilariae by *culex quinquefasciatus*: A study under natural conditions. Parasitology 116(03): 243-255.

19. Ho BC, Ewert A. (1967) Experimental transmission of filarial larvae in relation to feeding behaviour of the mosquito vectors. Transactions of the Royal Society of Tropical Medicine and Hygiene 61(5): 663-666.

20. May RM. (1977) Togetherness among schistosomes: Its effects on the dynamics of the infection. Mathematical Biosciences 35(3–4): 301-343.

21. Duerr HP, Dietz K, Eichner M. (2005) Determinants of the eradicability of filarial infections: A conceptual approach. Trends in Parasitology 21(2): 88-96.

LYMFASIM model description

The simulation model, LYMFASIM, describes the transmission and control of lymphatic filariasis in a dynamically changing human population [1]. The model employs the technique of stochastic microsimulation [2]. The model describes the dynamics of human, vector, and the parasites. The microsimulation technique allows LYMFASIM to take account of variation in in the human population characteristics (age, sex, exposure to mosquito bites, ability to develop immune responses, inclination to get treatment, etc) and in the adult parasites (lifespan, production of Mf, etc). Details of parasite control (coverage, frequency and duration of treatment, drug effects on adult parasites and/or microfilaria), vector control (duration, effect of vector control in terms of reduction in man vector contact), and surveillance (time and type of survey, whether the complete population is examined or a selected cohort, diagnostic test characteristic including the volume of the blood sample for mf detection) can be specified. A detailed description and mathematical formulation of the model are given in an earlier publication [1]. Here we provide a brief description.

Human population dynamics

The human population dynamics is governed by birth and death processes. The probability to die at a certain age is defined by a life table. The cumulative death rate for intermediate ages is obtained by linear interpolation. The expected number of births (per year) at a given moment depends on the number of women and their age-distribution. Immigration and emigration are not considered in the model.

As explained above, the model allows for heterogeneities within the human and parasite population. The characteristics of individuals are determined by chance and determine an individual's infection load in the absence of interventions. The model keeps track of the life histories of each human individual, focusing on the acquisition and loss of adult worms, microfilaria density and participation in mass treatment (further explained below).

Transmission and parasite dynamics

A key-variable in the model is the force-of-infection, *foi*, defined as the number of new immature parasites (i.e., those that survived the larval stage) per month. This *foi* will vary over time and between individuals. For individual *i* at time *t*, it is calculated as follows:

$$foi_i(t) = mtp_i(t) \times sr \times [1 - Rl_i(t)]$$

The variable *Rl* describes the level of immunity to L3-larvae and may vary between 0 (no immunity) and 1 (full immunity, no L3-larva will survive). Its calculation will be presented below. The parameter *sr* describes the chance that an inoculated L3 larva will survive and reach the stage of immature worm in the absence of immunity ('success-ratio'). The variable *mtp* is the monthly transmission potential, which follows from:

$$mtp_i(t) = mbr(t) \times \overline{L3}(t) \times E_i(t)$$

with *mbr(t)* quantifying the monthly biting rate (no. of mosquito-bites per month) for an average adult person, $\overline{L3}(t)$ being the average number of infective larvae released per vector-bite and $E_i(t)$ quantifying a person's relative exposure. All these variables are time-dependent. The relative exposure of a person has two components: an age-sex dependent component (described by the function $Ea_i(a(t),s)$) and a random component ('exposure index', *Ei*):

$$E_i(t) = Ea_i(a_i(t), s_i) \times Ei_i$$

In many endemic areas the prevalence and intensity of infection among males is higher than among females [3], which could reflect sex-differences in exposure. However, in order to reduce the complexity of the model, in this paper we assume an equal exposure between the sexes. Possible sex-differences are assumed to be included in random exposure variation (E_i). The age dependent component is described as follows: at birth a person has an exposure of E_0, thereafter it increases linearly to reach a maximum of 1.0 at the age of a_{max}. The exposure index (random component) is assumed to be a life-long property of a person. Its value is randomly selected from a gamma probability distribution with mean = 1 and shape-parameter α_E. This gamma-distribution allows for persons with low or very low relative exposure.

The average number of infective larvae released per mosquito-bite ($\overline{L3}(t)$) is calculated as follows:

$$\overline{L3}(t) = v \times \frac{\sum_{i=1}^{N(t)} E_i(t) \times L3_i(t)}{\sum_{i=1}^{N(t)} E_i(t)}$$

In this expression *N* is the total number of individuals in the human population. $L3_i$ is the average number of L3-larvae resulting from a blood meal on person *i* given survival of the mosquito between the uptake of microfilariae and the development to the L3-stage. The linear factor *v* (<1) combines such factors like the probability that an L3-larva will be released, the fraction of potentially infectious mosquitoes (i.e., those that had a blood meal before), and survival of the mosquitoes under field conditions.

Based on experimental data, the relation between the human Mf density *m* (as measured by the number of Mf in a blood-smear of 20 μl) and the number of L3 that will develop in feeding mosquitoes (given survival of the mosquitoes) is described by the following hyperbolic function [4]:

$$L3_i(t) = \frac{\varphi \times m_i(t)}{1 + \zeta \times m_i(t)}$$

This relationship saturates at φ/ζ at high human Mf densities and has an initial slope of φ. Because of this saturation, the development of the parasite in the vector is one of the density regulation mechanisms in the transmission of the parasite.

In case of a constant *foi* (which may be approximated in adults and in the absence of interventions or strong natural fluctuations), the expected equilibrium number of adult worms equals:

$$M_i = foi_i \times (\overline{Tl} - Ti)$$

$\overline{Tl}$ is the mean lifespan of the parasite and *Ti* the duration of the immature stage. The lifespan is assumed to vary between parasites according to a Weibull distribution with mean $\overline{Tl}$ and shape-parameter α_{Tl}. The duration of the immature stage is considered to be invariable. The parasite lifespan not only determines the equilibrium worm-load, but also the rate at which the worm-load declines in case of interruption of transmission.

The dynamics in the Mf density *m* is given by the following expression:

$$m_i(t) = s \times m_i(t-1) + F_i(t) \times r_i(t)$$

with *s* being the monthly survival of the microfilariae and *r* the number of microfilariae produced by each female worm per month and per 20 μl peripheral blood taken for diagnosis. *F* quantifies the number of adult female worms. On average $F=M/2$, but because the worm-load *M* is a discrete number, particularly at low worm numbers the sex-ratio may deviate from 1:1. The Mf density is defined as the average number of Mf in a 20-μl blood smear or a multiplication of this. The actual (discrete) number of Mf counted in the smear is a random selection from a Negative Binomial distribution with mean = m_i and parameter of dispersion k_m.

The Mf production *r* follows from:

$$r_i(t) = r_0 \times [1 - Rw_i(t)]$$

In this expression r_0 is the Mf production in the absence of an immune-response and in the presence of at least one adult male in the human host. In case of single-sex infections, Mf production is assumed to be

zero. *Rw* quantifies the immune-responsiveness against the adult worms (see below). In case *Rw*=1, the immune-system will completely block the development and production of microfilariae.

In LYMFASIM we assume two mechanisms for the impact of the immune system on the dynamics of the parasite: (1) anti-L3 immunity caused by prolonged exposure to L3-antigens, resulting in a reduction of the success of inoculated L3-larvae to mature and settle in the human body, and (2) anti-worm immunity caused by the cumulative presence of adult parasites, modelled as a reduction in the fecundity of female worms. Though the way it is modelled in LYMFASIM is debatable, the assumption of these two types of responses is in accordance with the existing knowledge about the regulation of the parasite [5, 6].

The first type of response is deduced from the work of Day *et al.* [7] who found antibodies to the L3 surface mainly in subjects of 20 years and older, i.e. subjects with the longest history of L3-inoculation. An age-specific increase in the presence of antibodies was also found by Beuria *et al*. [8], who further concluded that antibody levels were highly variable between individuals. As regards the anti-worm immunity, it has been suggested that prolonged presence of adult parasites causes a breakdown in tolerance to the parasites, resulting in clearance of microfilariae and progress of disease [5]. Whether and to what extent the adult worms or the microfilariae are the target of this response is not clear. In the model we assume that immune-responses cause a reduced fecundity (Mf output).

The mathematical formulation for both types of immune-responses is analogous to a model proposed by Woolhouse [9]. The level and dynamics of anti-L3 immunity in person *i* at time *t* is given by:

$$Rl_i(t) = 1 - \exp[-\gamma_l \times \rho_{l,i} \times Hl_i(t)]$$

$$Hl_i(t) = \beta_l \times Hl_i(t-1) + mtp_i(t)$$

The implication of the anti-L3 immunity *Rl* is given in equation 2. It varies with the ‘experience of L3 infection’, *Hl*, which increases with the number of inoculated L3-larvae (quantified by the monthly transmission potential *mtp*) and which decreases each month with a factor β_l. This decrease represents the decay in specific memory cells or antibodies. The factor γ_l (‘strength of anti-L3 immunity’) translates the experience of L3-infection into an immune-response. Noticing that the immune-responsiveness may vary between individuals even in the case of similar experience of infection, the factor ρ_l is included. This factor varies according to a gamma probability distribution with mean = 1 and shape-parameter α_l. This definition of anti-L3 immunity is analogous to the ‘LL’ model described by Woolhouse [9].

The expressions for the anti-worm (anti-fecundity) response are highly similar to those for the anti-L3 response and is analogous to Woolhouse’s ‘AE’ model:

$$Rw_i(t) = 1 - \exp[-\gamma_w \times \rho_{w,i} \times Hw_i(t)]$$

$$Hw_i(t) = \beta_w \times Hw_i(t-1) + M_i(t)$$

This type of response is driven by the current and past worm-load *M* (experience of worm-infection). The factors β_w (decay of specific antibodies of memory cells), γ_w ('strength of anti-worm immunity'), and ρ_w (immunity variation; described by a gamma distribution with mean 1 and shape α_w) are thought to be independent from the corresponding factors for the anti-L3 immunity. The impact of anti-worm immunity is given by equation 9.

The factors β_l and β_w determine the duration of the immune-responses after cessation of boosting (inoculation of L3-larvae, presence of worms). In the remainder, we will consider the following transformation of these parameters:

$$THl = [\ln(0.5)/\ln(\beta_l)]/12$$

$$THw = [\ln(0.5)/\ln(\beta_w)]/12$$

These transformations express the half-life (in years) of the experience of infection (*Hl* or *Hw*) in the absence of boosting. We will call this 'immunological memory'.

A complete list of all model assumptions and parameter values is provided in Table S2, with specification of their source.

Table S2. LYMFASIM input: probability distributions, functions and parameter values.

Parameter description (symbol)	Model variant for India (Pondicherry)		Model variant for Africa (Malindi)		Model variant for PNG (Nanaha)		
**Human demography**							
Cumulative survival (F(a)), by age	Age	Survival	Age	Survival	Age	Survival	Fixed, as in [10, 11]
	0	1	0	1	0	1	
	5	0.904	5	0.804	5	0.804	
	10	0.895	15	0.78	15	0.78	
	15	0.888	20	0.755	20	0.755	
	20	0.879	25	0.73	25	0.73	
	25	0.864	30	0.707	30	0.707	
	30	0.849	35	0.654	35	0.654	
	40	0.812	40	0.605	40	0.605	
	50	0.756	45	0.56	45	0.56	
	90	0	50	0.506	50	0.506	
			60	0.407	60	0.407	
			70	0.255	70	0.255	
			80	0.051	80	0.051	
			90	0	90	0	
Fertility rate per woman (R(a)), by age	Age	Fertility rate	Age	Fertility rate	Age	Fertility rate	Fixed, as in [10, 11]
	0	0	0	0	0	0	
	5	0	5	0	5	0	
	10	0	15	0	15	0	
	15	0	20	0.116	20	0.116	

	20	0.075	25	0.23	25	0.23	
	25	0.254	30	0.245	30	0.245	
	30	0.222	35	0.207	35	0.207	
	40	0.096	40	0.147	40	0.147	
	50	0.013	45	0.077	45	0.077	
	90	0	50	0.031	50	0.031	
			60	0	60	0	
			70	0	70	0	
			80	0	80	0	
			90	0	90	0	
Initial population	Age	Males/females	age	Male/females	age	Male/females	Fixed, as in [10, 11]
	5	20/20	5	20/20	5	20/20	
	10	17/17	15	31/31	15	31/31	
	15	15/15	20	12/12	20	12/12	
	20	15/15	25	11/11	25	11/11	
	25	22/22	30	10/10	30	10/10	
	30	20/20	35	8/8	35	8/8	
	40	15/15	40	7/7	40	7/7	
	50	13/13	45	5/5	45	5/5	
	90	13/13	50	4/4	50	4/4	
			60	7/7	60	7/7	
			70	4/4	70	4/4	
			80	1/1	80	1/1	
			90	1/1	90	1/1	
Maximum population size	5000		750		750		Assumed
Proportion removed when maximum population size is reached	10%		10%		10%		Assumed

Exposure

External force-of-infection at start of burn-in period	0.5	2	2	Assumed
Duration of external force-of-infection at start of burn-in period	4 years	2 years	2 years	Assumed
Average mosquito biting rate for adult men (mbr)	fitted to data	fitted to data	fitted to data	
Seasonal variation	no	no	no	
exposure at age 0 (E0)	0.26	0	0	Previously estimated by fitting to data [10];slightly adjusted for africa/PG
age at which maximum exposure is reached (amax)	19.1	20	20	Previously estimated by fitting to data [10];slightly adjusted for africa/PG
Type of probability distribution describing variation in the individual exposure index Ei, due to personal factors (fixed through life) given age and sex	gamma distribution	gamma distribution	gamma distribution	Assumed
mean	1	1	1	By definition
α_E	1.13	0.26	fitted to data	Previously estimated

				by fitting to data [10, 11]
**Parasite dynamics within host**				
Success ratio (sr)	0.00103	0.00088	0.00088	Previously estimated by fitting to data [10, 11]
Shape-parameter for the gamma-distribution describing individual variation in the ability to develop an anti-L3 immune-response (rho-l)	1.07	NA	NA	Previously estimated by fitting to data [10]
Strength of immunological memory for anti-L3 immunity (gamma-l)	0.0000589	NA	NA	Previously estimated by fitting to data [10]
Duration of immunological memory for anti-L3 immunity (THl), in years	9.6	NA	NA	Previously estimated by fitting to data [10]
Anti-fecundity immunity				
Shape-parameter for the gamma-distribution describing individual variation in the ability to develop an anti-L3 immune-response (rho-w)	NA			
Strength of immunological memory for anti-L3 immunity (gamma-w)	NA			
Duration of immunological memory for anti-L3 immunity (THw), in years	NA			

Average worm lifespan (Tl)	10.2 years	10.2 years	10.2 years	Previously estimated by fitting to data [10]
Type of distribution	Weibull	Weibull	Weibull	Fixed, based on the estimated variation in adult worm lifepan for *Onchocerca volvulus* [12, 13]
α_{Tl}.	2	2	2	
Duration of immature stage of the parasite in human host (Ti)	8 months	8	8	Fixed [14]
No. of Mf produced per female parasite per month per 20 ml peripheral blood in the absence of immunereactions and in the presence of at least 1 male worm (r0)	0.606	0.58	0.58	
Monthly survival of the microfilariae, fraction (s)	0.9	0.9	0.9	Fixed, based on [15]
association between worm age and mf production rate	NA			
Polygamy (All female worms produce mf in the presence of at least one male worm.)	yes	yes	Yes	
**<u>Uptake of infection by the vector</u>**				
Function relationship	$L3_i(t) = \frac{\varphi \times m_i(t)}{1+\zeta \times m_i(t)}$	L3=a(1-exp (-(bM)^c)	L3=a(1-exp (-(bM)^c)	[4, 11]

	φ = 0.09,	a	1.666	a	1.666	
	ζ = 0.013	b	0.027	b	0.027	
		c	1.514	c	1.514	
Transmission probability (v), fraction of the L3 larvae, resulting from a single blood meal, that is released by a mosquito	0.1	0.1		0.1		Fixed, as in [10]
**<u>Other</u>**						
Duration of warming up period	131	152		144		Assumed
**<u>Surveillance</u>**						
Volume of blood examined for mf	20	100		200		Assumed
skin snip variability	0.345	1.65		3.3		Previously estimated for 20 µL blood by fitting to data [10]; assumed for Africa and PNG
**<u>Morbidity</u>**						
not applicable						
no excess mortality due to disease						

Control measures

Mass drug administration (MDA): The impact of MDA can be simulated by specifying the exact moments of treatment (year, month), the drug or administration regimen applied with its efficacy, the fraction of people treated per round (coverage), and the compliance pattern.

The model considers three possible effect mechanisms: (1) a fraction of adult worms is killed; (2) a fraction of female adult worms is permanently sterilized (i.e. they stop producing Mf); and (3) a fraction of mf is killed. The fraction of parasites affected can be constant or can vary according to a chosen probability distribution function.

The effect of treatment on Mf is described as follows:

$$m_i(t+\varepsilon) = m_i(t) \times (1 - dm_i)$$

with m_i the number of microfilariae (scaled to the 20 μL blood volume normally used for diagnosis) at time t, treatment occurring between time t and $t+\varepsilon$, and dm_i the fraction if Mf killed as a result of the treatment. The latter is stochastic variable (<= 1) which varies between persons and between treatments and which is described by a user-defined probability distribution function.

The effect of treatment on adult worms is described by:

$$M_i(t+\varepsilon) = M_i(t) \times (1 - dM_i)$$

with M_i the number of adult worm per individual and dM_i the fraction of worms killed as a result of the treatment. dM_i is again a stochastic variables (<= 1) which varies between persons and between treatments and which is described by a user-defined probability distribution function.

A permanent or temporary reduction in mf production $r_i(t)$ by female parasites in individual i, who was treated at time τ, is simulated as follows:

$$r_i(t) = r_i^{'}(t) \times (1 - df_i) \times \left(\frac{t-\tau}{Tr_i} \right)^{\sigma}, \text{ for } t-\tau < Tr_i$$

$$= r_i^{'}(t) \times (1 - df_i), \text{ otherwise}$$

With $r_i^{'}(t)$ being the Mf production of female worms had person i not been treated (the outcome of equation [9], df_i (<= 1) the irreversible reduction of fecundity caused by the treatment, Tr_i the period during which the female parasite recovers from treatment (assuming that immediately after treatment production is zero), and σ (>0) a shape parameter which determines how Mf production increases during

the recovery period Tr: σ=1 implies a linear increase, σ <1 implies that recovery of productivity is mainly at the end of the period Tr (note that $\sigma \rightarrow 0$ is equivalent to $Tr \rightarrow 0$, while $\sigma \rightarrow \infty$ implies the total absence of Mf prodution during Tr).Both Tr_i and df_i are stochastic variables described by a probability distribution function.

All stochastic variables related to the effects of treatment (*dm*, *dM*, *df*, *Tr*) are by default assumed to be independent and to be generated for each person at each treatment. (The user can also to chose to attribute treatment efficacy as a fixed characteristic to an individual, who in that case always responds in the same way to treatment.)

The compliance pattern describes the tendency of persons to participate in repeated treatment rounds. In case of random compliance, all individuals have the same probability to be treated (equal to the fraction covered). In case of systematic compliance, each person in the population is characterized by an invariable compliance factor (a random number between 0 and 1), which results in a treatment probability of either 1 (for compliance factor ≤ coverage) or 0 (for compliance factor> coverage). Consequently, if coverage is constant over time, some individuals will always be treated while the remaining persons are never treated. In the case of semi-systematic compliance pattern, the compliance factor indicates a person's tendency to participate. Random numbers define whether an individual is actually treated or not. The latter pattern is presumably most realistic [16].

LYMFASIM also allows the simulation of selective treatment. In that case, treatment is only provided to those persons who were Mf positive in the most recent survey (which may take place in the same month as treatment, see below). Coverage and compliance play no role.

Vector control: Vector control is modelled as a reduction of the average monthly biting rates during a given period of time. The number of such periods can be chosen. A period of vector control is specified by the year + month of the beginning of the strategy and the year + month of the end of a strategy.

Epidemiological Surveys

During the simulation, surveys will take place at user-defined time points. During a survey, for all simulated individuals, the actual number of male and female worms is recorded, and a diagnostic test is simulated to obtain Mf counts. It is assumed that Mf are counted in 20μL blood smear, or a multiple φ of this (taken at the appropriate time during the day in view of circadian periodical appearance in the blood). The Mf count Mf_i for person i is then given by:

$$Mf_i = Negbin(\phi \times m_i, k_m)$$

i.e. the number is assumed to follow a negative binomial distribution with clumping factor k_m. The user can specify other distributions if deemed more appropriate.

Model outcomes

The primary outcomes of the model are predicted trends in the Mf prevalence and mean Mf intensity in the population as well as age-and gender specific prevalence and intensity. These outcomes are based on Mf counts for all individuals in the population, while taking account of test characteristics that determine sampling variation and the possibility of false-negative test results. This makes simulation outcomes directly comparable to field data. For the present study, we assumed that Mf counts were done by microscopic examination of a 20-ml or 60-ml thick smear of night finger-prick blood.

Approach to model fitting

We fitted the model to community-specific age-patterns in mf prevalence, based on a chi-square goodness-of-fit statistics (see below). In the following, we discuss the details of the fit procedure per location:

- Pondicherry, India (vector *Culex quinquefasciatus*)
  - We fixed all model parameters at the previously derived point estimates, except the monthly biting rate (MBR). See Subramanian et al 2004 [10] for a complete overview of all parameter values.
  - Model predicted mf prevalence were derived assuming that mf are counted in one 20μL blood smear.
  - The MBR was fitted to data. We assumed no seasonality in transmission, so that the MBR is equal for all 12 months and ABR = 12 * MBR. We performed a large number of simulations, varying the MBR biting rate from 1500 to 3500 with increments of 10, doing multiple repeated runs for each biting rate value. Outcomes of repeated runs with the same MBR vary, due to stochasticity in the model. In total we did about 10,000 simulations.
  - For each individual run (defined by MBR and seed for the random number generator) we calculate the chi-square, as indicator for the goodness-of-fit of model predictions to baseline data.
  - We identified MBR and seed combinations that resulted in a good fit to the baseline data and went on to predict trends infection during integrated vector management.
  - NB. The point estimates of fixed model-parameters were estimated based on a much more extensive analysis of longitudinal data from the same location. See Subramanian et al 2004 [10]for a complete description of methods and overview of all parameter values.
- Malindi, Kenya (vector *anopheles*):
  - We fixed all model parameters at the previously derived point estimates for our Africa model, except for the MBR. See Stolk et al 2008 [11] for an overview of all parameter values.

- Model predicted mf prevalence were derived assuming that mf are counted in five 20μL blood smear, 100 μL in total.
- We fitted the MBR to data as we described above for Pondicherry. Again, we assumed no seasonality in transmission, so that the MBR is equal for all 12 months and ABR = 12 * MBR. We performed a large number of simulations, varying the MBR biting rate from 400 to 3000 with increments of 10, doing multiple repeated runs for each biting rate value. Outcomes of repeated runs with the same MBR vary, due to stochasticity in the model. In total we did about 10,000 simulations.
- For each individual run (defined by MBR and seed for the random number generator) we calculate the chi-square, as indicator for the goodness-of-fit of model predictions to baseline data.
- We identified MBR and seed combinations that resulted in a good fit to the baseline data and went on to predict trends infection during MDA, with fixed input assumptions regarding drug efficacy and treatment coverage.

- Nanaha, Papua New Guinea (vector *anopheles*):
  - LYMFASIM was not previously quantified to mimick transmission dynamics is Papua New Guinea.
  - We assumed that the biological parameters are not different from the African regions with *Anopheles*-transmitted infection.Therefore, all parameters were kept the same as in the Africa model, except for the two parameters related to exposure patterns. See Stolk et al 2008 for an overview of all fixed parameter values.
  - The two fitted parameters were
    - MBR
    - The shape-parameter α_E of the gamma distribution, describing variation in exposure between individuals
  - To assess which combinations of MBR and α_E value are in concordance with observed data, we varied both parameters randomly (MBR was varied between 1 and 3500; and $1/\alpha_E$ was varied between 0 and 10). We did only one run per combination (always the same seed for the random number generator).
  - As described above, we used chi-square to assess the goodness-of-fit of model to data for each indiviual run (combination of MBR, α_E value). For each individual run (defined by MBR and seed for the random number generator) we calculate the chi-square, as indicator for the goodness-of-fit of model predictions to baseline data.
  - We identified MBR and α_E value that resulted in a good fit to the baseline data and went on to predict trends in infection during MDA, with fixed input assumptions regarding drug efficacy and treatment coverage.

Goodness of fit statistics

Simulation results are compared with the data using the following equation, for each age group separately:

$$\mathrm{X}^2 = \frac{\left(a1 - \frac{n1}{n}(a1 + a2)\right)^2}{\frac{n1}{n}(a1 + a2)} + \frac{\left(b1 - \frac{n1}{n}(b1 + b2)\right)^2}{\frac{n1}{n}(b1 + b2)} + \frac{\left(a2 - \frac{n2}{n}(a1 + a2)\right)^2}{\frac{n2}{n}(a1 + a2)} + \frac{\left(b2 - \frac{n2}{n}(b1 + b2)\right)^2}{\frac{n2}{n}(b1 + b2)}$$

Where,

a1= number of mf positives in the age group under consideration, in the data
b1= number of mf negatives in the age group under consideration, in the data
n1 = total number examined in the age group under consideration, in the data
a2= number of mf positives in the age group under consideration, in the model
b2= number of mf negatives in the age group under consideration, in the model
n2 = total number examined in the age group under consideration, in the model
n = n1+n2

The fit of model predictions to data was considered to be acceptable if the chi-square value for each age group was below 3.84.

Due to stochastic nature of the various processes involved in the model, the simulation output will be subject to random variation and will only represent an estimate of the true outcome of the model.

References

1. Plaisier AP, Subramanian S, Das PK, Souza W, Lapa T, Furtado AF et al. The LYMFASIM simulation program for modeling lymphatic filariasis and its control. Methods of Information in Medicine. 1998;37:97-108.
2. Habbema JDF, De Vlas SJ, Plaisier AP, Van Oortmarssen GJ. The microsimulation approach to epidemiologic modeling of helminthic infections, with special reference to schistosomiasis. Am J Trop Med Hyg. 1996;55(5 Suppl):165-9.
3. Brabin L. Sex differentials in susceptibility to lymphatic filariasis and implications for maternal child immunity. Epidemiol Infect. 1990;105(2):335-53.
4. Subramanian S, Krishnamoorthy K, Ramaiah KD, Habbema JDF, Das PK, Plaisier AP. The relationship between microfilarial load in the human host and uptake and development of *Wuchereria bancrofti* microfilariae by *Culex quinquefasciatus*: a study under natural conditions. Parasitology. 1998;116:243-55.
5. Maizels RM, Lawrence RA. Immunological tolerance: the key feature in human filariasis? Parasitology Today. 1991;7(10):271-6.
6. Ottesen EA. The Wellcome Trust Lecture. Infection and disease in lymphatic filariasis: an immunological perspective. Parasitology. 1992;104(Suppl):S71-9.
7. Day KP, Grenfell B, Spark R, Kazura JW, Alpers MP. Age specific patterns of change in the dynamics of *Wuchereria bancrofti* infection in Papua New Guinea. Am J Trop Med Hyg. 1991;44(5):518-27.
8. Beuria MK, Bal M, Dash AP, Das MK. Age-related prevalence of antibodies to infective larvae of *Wuchereria bancrofti* in normal individuals from a filaria-endemic region. J Biosci. 1995;20(2):167-74.
9. Woolhouse ME. A theoretical framework for the immunoepidemiology of helminth infection. Parasite Immunol. 1992;14(6):563-78.
10. Subramanian S, Stolk WA, Ramaiah KD, Plaisier AP, Krishnamoorthy K, Van Oortmarssen GJ et al. The dynamics of *Wuchereria bancrofti* infection: a model-based analysis of longitudinal data from Pondicherry, India. Parasitology. 2004;128(Pt 5):467-82.
11. Stolk WA, de Vlas SJ, Borsboom GJ, Habbema JD. LYMFASIM, a simulation model for predicting the impact of lymphatic filariasis control: quantification for African villages. Parasitology. 2008;135(13):1583-98.
12. Plaisier AP, van Oortmarssen GJ, Remme J, Habbema JD. The reproductive lifespan of *Onchocerca volvulus* in West African savanna. Acta Trop. 1991;48(4):271-84.
13. Remme J, De Sole G, van Oortmarssen GJ. The predicted and observed decline in onchocerciasis infection during 14 years of successful control of Simulium spp. in west Africa. Bull World Health Organ. 1990;68(3):331-9.
14. World Health Organization. Lymphatic filariasis: the disease and its control. Fifth report of the WHO Expert Committee on Filariasis. World Health Organ Tech Rep Ser. 1992;821:1-71.
15. Plaisier AP, Cao WC, van Oortmarssen GJ, Habbema JD. Efficacy of ivermectin in the treatment of *Wuchereria bancrofti* infection: a model-based analysis of trial results. Parasitology. 1999;119(Pt 4):385-94.
16. Plaisier AP, Stolk WA, van Oortmarssen GJ, Habbema JD. Effectiveness of annual ivermectin treatment for *Wuchereria bancrofti* infection. Parasitol Today. 2000;16(7):298-302.

TRANSFIL model description

The TRANSFIL model of LF infection was employed [6]. The model is a multi-scale stochastic simulation of individuals with worm burden, microfilaraemia and other demographic parameters relating to age and risk of exposure. Humans are modelled individually, with their own male and female worm burden denoted W_i^m and W_i^f. The density of mf in the peripheral blood is also modelled for each individual and denoted M_i . The total mf density in the population contributes towards the current density of L3 larvae in the human-biting mosquito population. The model dynamics are divided into the individual human dynamics, including age and turnover; worm dynamics inside the host; microfilariae dynamics inside the host and larvae dynamics inside the mosquito.

Worm dynamics

For each individual i both male and female worms are added according to their bite risk b_i that is individually drawn from a gamma distribution with mean 1 and shape parameter k. The rate at which worms are acquired depends on a number of stages of larvae life cycle as well as characteristics of the host. These are the mean total number of bites per individual per month b_r; the probability that an L3 larvae leaves the host during a biting event ψ_1; the probability that the L3 enters the host ψ_2; the proportion of L3 within the host that develop into adult worms; the age-dependent biting rate $h(a)$ that increases with body size to saturate at age nine [8]; and the mean equilibrium larvae density L^*. Each worm has a constant rate of death μ, which is the same for males and females. The stochastic dynamics for an individual i can be summarised as,

$$W_i^m \to W_i^m + 1 \text{ at rate } 0.5 b_r b_i \psi_1 \psi_2 s_2 h(a_i) L^*, \tag{1a}$$

$$W_i^f \to W_i^f + 1 \text{ at rate } 0.5 b_r b_i \psi_1 \psi_2 s_2 h(a_i) L^*, \tag{1b}$$

$$W_i^m \to W_i^m - 1 \text{ at rate } \mu W_i^m, \tag{1c}$$

$$W_i^f \to W_i^f - 1 \text{ at rate } \mu W_i^f. \tag{1d}$$

Mf dynamics

The microfilariae dynamics are dependent on the total number of adult male and female worms. *W. bancrofti* is assumed to be completely polygamous [15] and hence the rate at which mf are produced is dependent upon the number of female worms combined with the presence of male worms. It is also assumed that there is death of the microfilariae that is constant and independent of the density of the mf. The dynamics of mf for an individual i are therefore

$$\frac{\mathrm{d}M_i}{\mathrm{d}t} = \alpha W_i^f \mathbb{I}(W_i^m > 0) - \gamma M_i \tag{2}$$

where the function $\mathbb{I}$ is one if there are male worms and zero if not.

Larvae dynamics

The larvae develop from the mf that enter the mosquito during a blood meal from an infected host. There are two functional forms of this relationship that differ according to mosquito genus. For *Culex*, where the cibarial armature is less-developed than in other species, mf can survive at lower densities [7]. The relationship here is

$$L(m) = \kappa_{s1}(1 - e^{-r_1 m/\kappa_{s1}}).$$

For *Anopheles* there is a facilitation relationship with limitation for larger concentration of host mf. This relationship is given by

$$L(m) = \kappa_{s2}\left(1 - e^{-r_2 m/\kappa_{s2}}\right)^2,$$

where m here is concentration of mf per 20μL taken during a blood meal and r, κ are parameters relating to the functional form of the uptake curve [3]. Each individual contributes towards the pool of larvae in the mosquito population according to their concentration of mf in the peripheral blood along with their intrinsic bite-risk b_i. The uptake of larvae is an average of all individual's mf concentration weighted by their bite-risk i.e.

$$\tilde{L} = \sum_i L(m_i)b_i / \sum_i b_i,$$

giving the average number of larvae per mosquito.

The dynamics of larvae in the mosquito are fast compared with the other aspects of infection due to the relatively short life-span of mosquitoes compared with filarial worms. The density is dependent on the number of bites per mosquito λ, the proportion of mosquitoes which pick up infection when biting an infected host g; the death rate of mosquitoes σ and the proportion of L3 leaving the mosquito per bite, ψ. The averaged number of larvae taken up in the population $\tilde{L}$ is calculated from the uptake curves described above,

$$\frac{\mathrm{d}L}{\mathrm{dt}} = \lambda g \tilde{L} - (\sigma_1 + \lambda\psi_1)L.$$

Finally the equilibrium value for L3 in a mosquito is given by

$$L^* = \frac{\lambda g \tilde{L}}{\sigma_1 + \lambda\psi_1},$$

Host dynamics

Each individual begins with zero infection and a bite-rate exposure that is drawn from a Gamma-distribution with mean 1 and shape parameter k. The shape parameter defines how aggregated bites are amongst individuals and consequently defines the aggregation of infection amongst individuals. The human death rate τ is assumed to be constant throughout an individual's lifetime with a cut-off at age 100. This results in an exponential with cut-off distribution for the ages of individuals in a simulation.

Modelling of intervention

MDA

An MDA event occurs simultaneously across all individuals. Before the start of a campaign, each individual is assigned a random value u_i defining their likelihood of participating in an MDA round. These u_i are drawn from a distribution that controls for both the coverage of MDA and the correlation between rounds (systematic non-adherence) (see [6] for details). At an MDA event a random variable z is drawn for each individual and if $z > u_i$, then that individual receives treatment. This means that the pre-defined coverage is an average and the actual coverage in a simulation will fluctuate around this average.

For an individual that receives MDA, their mf concentration (M_i) and their male and female worm burden (W_i^m and W_i^f) are reduced by a factor according to the efficacy of the treatment. furthermore there is a period after MDA during which the production of mf for that individual is diminished.

Vector control

Vector control is considered via integrated vector management (IVM). For the period under which IVM occurs, the average monthly bite rate b_r is reduced by a factor, which has been previously estimated.

Model fitting

We employed an Approximate Bayesian Computation (ABC) fitting approach with a Particle Control Rejection (PRC) scheme [18]. The main idea of ABC is to perform Bayesian inference when a likelihood is either computationally-intractable or not feasible to define. As an alternative, a sufficient summary

statistic is used for the model data and compared to the data to be fitted. A distance metric is used to define the error between the data drawn from the model and the real data. As the error between the summary statistics of the model-generated data and real data approaches zero, the posterior distribution is approximated with greater accuracy. More precisely, the function f summarises the data D in some form. Model data drawn from the parameters θ are given as M^*_θ, where the star denotes this is a realisation of the model-data and is subsequently a random variable. If ρ is a distance metric for the summary data, then the posterior is calculated as

$$P(\theta|D) \approx P(\rho(f(D), f(M^*_\theta)) < \epsilon), \tag{3}$$

where the error in the approximation reduces as ϵ is reduced.

The basic algorithm is as follows. For all parameters that are to be fitted (θ), a number of particles (samples) are drawn from the prior distribution $P(\theta)$ to produce a set of particles θ^*_i. A pre-defined set of tolerances $\{\epsilon_0, \ldots, \epsilon_T\}$ is used to allow exploration or the parameter space as well as avoiding particles becoming stuck in local minima. A new set of particles is generated by randomly sampling from θ^*_i and perturbed using a zero-mean Gaussian random variable with small variance. The newly generated particle is accepted if $\rho(f(D), f(M^*_\theta)) < \epsilon$, else it is rejected and another particle is generated according the procedure defined. Once the desired number of particles has been accepted the tolerance is lowered and a new set of particles are generated as before. Once the particles are generated for the smallest tolerance (ϵ_T), the algorithm terminates and these are used as the sample for the posterior.

For the fitting procedure, the summary statistic used was the age-prevalence for the age categories $[< 10, 10-19, 20-29, 30-39, 40-49, 50-59, 60-70]$. The distance metric ρ used was a weighted sum of squares $\sum_i w_i(p_i - p^*_i)^2$. The weights w_i are the total number of individuals in each age-category in the data, this was used to avoid over-fitting to age-classes that had few individuals in them.

Table 1: Summary of parameter values used to inform the model.

**Parameter symbol**	**Definition**	**Value**	**Source**
λ	Number of bites per mosquito	10 per month	[10, 13]
V/H	Ratio of number of vectors to hosts	fitted to baseline data	N/A
$a_{\max}$	Age at which exposure to mosquitoes reaches its maximum level	20.0	[15]
ψ_1	Proportion of L3 leaving mosquito per bite	0.414	[4]
ψ_2	Proportion of L3 leaving mosquito that enter host	0.32	[5]
s_2	Proportion of L3 entering host that develop into adult worms	fitted to baseline data	N/A
μ	Death rate of adult worms	0.0104 per month	[2, 9, 17, 16]
α	Production rate of mf per worm	0.2 per month	[4]
γ	Death rate of mf	0.1 per month	[4, 9]
g	proportion of mosquitoes which pick up infection when biting an infected host	0.37	[12]
σ	Death rate of mosquitoes	5 per month	[5]
k	Aggregation parameter of individual exposure to mosquitoes	fitted to baseline data	[14, 1]
$h(a)$	Parameter to adjust rate at which individuals of age a are bitten	linear from 0 to 10, with maximum of 1.	[8]
ρ	systematic adherence of MDA	0.25	[11]
p_C	coverage of MDA	varied	N/A

References

[1] PK Das, A Manoharan, S Subramanian, KD Ramaiah, SP Pani, AR Rajavel, and PK Rajagopalan. Bancroftian filariasis in pondicherry, south india–epidemiological impact of recovery of the vector population. *Epidemiology and infection*, 108(03):483–493, 1992.

[2] David B Evans, Hellen Gelband, and Carol Vlassoff. Social and economic factors and the control of lymphatic filariasis: a review. *Acta tropica*, 53(1):1–26, 1993.

[3] Manoj Gambhir and Edwin Michael. Complex ecological dynamics and eradicability of the vector borne macroparasitic disease, lymphatic filariasis. *PLoS One*, 3(8):e2874, 2008.

[4] Nelson G Hairston and Botha de Meillon. On the inefficiency of transmission of wuchereria bancrofti from mosquito to human host. *Bulletin of the World Health Organization*, 38(6):935, 1968.

[5] Beng C Ho and Adam Ewert. Experimental transmission of filarial larvae in relation to feeding behaviour of the mosquito vectors. *Transactions of the Royal Society of tropical Medicine and Hygiene*, 61(5):663–666, 1967.

[6] Michael Alastair Irvine, Lisa J Reimer, Sammy M Njenga, Sharmini Gunawardena, Louise Kelly-Hope, M Bockarie, and T Déirdre Hollingsworth. Modelling strategies to break transmission of lymphatic filariasis-aggregation, adherence and vector competence greatly alter elimination. *Parasites & vectors*, 8(1):1–19, 2015.

[7] Edwin Michael, Lucy C Snow, and Moses J Bockarie. Ecological meta-analysis of density-dependent processes in the transmission of lymphatic filariasis: survival of infected vectors. *Journal of medical entomology*, 46(4):873–880, 2009.

[8] RA Norman, Man-Suen Chan, A Srividya, SP Pani, Kapa D Ramaiah, Perumal Vanamail, Edwin Michael, Pradeep K Das, and Don AP Bundy. Epifil: the development of an age-structured model for describing the transmission dynamics and control of lymphatic filariasis. *Epidemiology and Infection*, 124(03):529–541, 2000.

[9] EA Ottesen and CP Ramachandran. Lymphatic filariasis infection and disease: control strategies. *Parasitology Today*, 11(4):129–130, 1995.

[10] PK RAJAGOPALAN. Population dynamics of culex pipiens fatigans, the filariasis vector, in pondicherry: Influence of climate and environment. *Proc. Indian natn. Sci. Acad. B46 No*, 6:745–752, 1980.

[11] Kathryn V Shuford, Hugo C Turner, and Roy M Anderson. Compliance with anthelmintic treatment in the neglected tropical diseases control programmes: a systematic review. *Parasites & vectors*, 9(1):1, 2016.

[12] S Subramanian, K Krishnamoorthy, KD Ramaiah, JDF Habbema, PK Das, and AP Plaisier. The relationship between microfilarial load in the human host and uptake and development of wuchereria bancrofti microfilariae by culex quinquefasciatus: a study under natural conditions. *Parasitology*, 116(03):243–255, 1998.

[13] S Subramanian, A Manoharan, KD Ramaiah, and PK Das. Rates of acquisition and loss of wuchereria bancrofti infection in culex quinquefasciatus. *The American journal of tropical medicine and hygiene*, 51(2):244–249, 1994.

[14] S Subramanian, SP Pani, PK Das, and PK Rajagopalan. Bancroftian filariasis in pondicherry, south india: 2. epidemiological evaluation of the effect of vector control. *Epidemiology and infection*, 103(03):693–702, 1989.

[15] S Subramanian, WA Stolk, KD Ramaiah, AP Plaisier, K Krishnamoorthy, GJ Van Oortmarssen, D Dominic Amalraj, JDF Habbema, and PK Das. The dynamics of wuchereria bancrofti infection: a model-based analysis of longitudinal data from pondicherry, india. *Parasitology*, 128(05):467–482, 2004.

[16] P Vanamail, KD Ramaiah, SP Pani, PK Das, BT Grenfell, and DAP Bundy. Estimation of the fecund life span of wuchereria bancrofti in an endemic area. *Transactions of the Royal Society of Tropical Medicine and Hygiene*, 90(2):119–121, 1996.

[17] P Vanamail, S Subramanian, PK Das, SP Pani, and PK Rajagopalan. Estimation of fecundic life span of wuchereria bancrofti from longitudinal study of human infection in an endemic area of pondicherry (south india). *The Indian journal of medical research*, 91:293–297, 1990.

[18] David M Walker, David Allingham, Heung Wing Joseph Lee, and Michael Small. Parameter inference in small world network disease models with approximate bayesian computational methods. *Physica A: Statistical Mechanics and its Applications*, 389(3):540–548, 2010.

B. Inter-model comparison of key parameters and functions

As an accompaniment to the full model descriptions found in the Supplementary Information (SI) and Table 1 in the main text, Table S4 captures the key similarities and differences in terms of the model parameters used and optimized during model induction and data fitting, and in running simulations of interventions using annual mass drug administrations (MDAs) and vector control. The implementation differences among the three models are highlighted here by drawing attention to the deterministic versus stochastic approaches in managing uncertainty. EPIFIL is a deterministic model which allows for variation by sampling the majority of its parameters from uniform prior distributions. LYMFASIM and TRANSFIL fix many of their parameters and incorporate variation through stochastic model elements.

Table S4 - Parameters and functions used in the LF models: EPIFIL (E), LYMFASIM (L), and TRANSFIL (T).

Component	Parameter/exogenous variable	@E	@L	@T	Refs
Humans	Pre-patency period	2	1	-	[1, 2]
	Worm mortality rate	2	1	1	[2-9]
	Production rate of microfilariae per worm	2	1	1	[2, 7-10]
	Death rate of the microfilariae	2	1	1	[2, 7-10]
	Proportion of mosquitoes which pick up infection when biting an infected host	2	-	1	[8, 9, 11]
	Maximum level of L3 given mf density	2	1	-	[2, 8, 9]
	Immunity waning rate	2	1	-	[2, 8, 9]
	The linear rate of increase in the aggregation parameter defined above	2	-	-	[8, 9, 12, 13]
	The establishment rate[a]	2	1	-	[2, 7-9, 14]
	Gradient of mf uptake[b]	2	1	-	[2, 8, 9]
	Strength of acquired immunity	2	1	-	[2, 8, 9]
	Strength of immunosuppression[c]	2	-	-	[8, 9]
	Slope of immunosuppression function[d]	2	-	-	[8, 9]
	Probability that an individual is of age	2	-	-	[8, 9, 15]
	Worm mating probability	2	-	-	[7-9, 16]
	Immunity to larval establishment	2	1	-	[2, 8, 9]
	Host immune suppression	2	-	-	[8, 9]
Vectors	Number of bites per mosquito	2	- (Implicit)	-	[7-9, 17, 18]
	Proportion of L3 leaving mosquito per bite	2	1	1	[2, 10]
	Death rate of mosquitoes	2	- (Implicit)	1	[8, 9, 14]
Community	Location parameter (*k*) of negative binomial distribution used in aggregation parameter	2	1/2	2	[2, 8, 9, 12, 13]
	Ratio of number of vector to hosts	1/2	2	2	Data
	Exposure to mosquito bites as function of age	2	1	1	Data
Intervention parameters	Proportion of mf killed for an individual MDA round	1	1	1	[2, 9, 19, 20]
	Proportion of worm killed for an individual MDA round	1	1	1	[2, 9, 19, 20]
	Period during which MDA survived adult worms do not reproduce	1	1	1	[2, 9, 19, 20]
	Systematic non-adherence of MDA	-	1	1	
	Coverage of MDA	varied	varied	varied	
	Reduction in the monthly biting rate owing to IVM (integrated vector management), relevant for the Pondicherry site only.	1	1	1	[2, 12, 13]

[a]The proportion of L3-stage larvae infecting human hosts that survive to develop into adult worms [8].
[b]The gradient of mf uptake *r* is a measure of the initial increase in the infective L3 larvae uptake by vector as *M* increases from 0 [8, 15].
[c] The facilitated establishment rate of adult worms due to parasite-induced immunosuppression in a heavily infected human host
[d] The initial rate of increase by which the strength of immunosuppression is achieved as *W* increases from 0[21].
@1: chosen from literature and used as fixed constant, 2: chosen from literature/expert knowledge and fitted.
(-): Not applicable

C. Performance-based model weighting

The construction of multi-model ensembles for simulating LF transmission in this study first required the calibration and validation of the three individual models (EPIFIL, LYMFASIM, and TRANSFIL) for each given data set. The three sets of single-model outputs were then combined using a mean-squared error performance-based weighting scheme which is described in detail below [22].

Given y_i, the observed baseline prevalence in age group *i*, a performance-based weighting for model *k* can be estimated as follows. First, we calculate the mean squared error (*MSE*) over N_k model simulations for model *k*:

$$MSE_k = \frac{1}{N_k I} \sum_{n=1}^{N_k} \sum_{i=1}^{I} (x_{kni} - y_i)^2,$$

where x_{kni} is the model-predicted prevalence in age group *i* for simulation *n* of model *k*. The weight for model *k*, denoted as w_k, is then calculated as the inverse of the *MSE*: $w_k = \frac{1}{MSE_k}$.

Further, individual normalized model weights for each of the *K* models can be calculated such that $\sum \hat{w}_k = 1$. This yields the following formula for the normalized weight:

$$\hat{w}_k = \frac{w_k}{\sum_{k=1}^{K} w_k},$$

The normalized weights are then applied to the single-model predictions such that the multi-model ensemble mean prediction for each age-group *i* is a weighted average of the three single-model predictions:

$$\bar{x}_{ki} = \frac{1}{N_k} \sum_{n=1}^{N_k} x_{kni}, \text{ and}$$

$$\bar{x}_{MM_i} = \sum_{k=1}^{K} \hat{w}_k \bar{x}_{ki},$$

The model weights can also be applied to the single-model variances to calculate the multi-model ensemble variance estimate which accounts for between-model and within-model variance.

$$\sigma_{ki}^2 = \frac{1}{N_k} \sum_{n=1}^{N_k} \left(x_{kni} - \bar{x}_{MM_i} \right)^2, \text{ and}$$

$$\sigma_{MM_i}^2 = \sum_{k=1}^{K} \hat{w}_k \sigma_{ki}^2,$$

In the above, $\bar{x}_{MM_i}$ denote the mean of the prediction in the ith age-group of the multi-model ensemble. The quantities σ_{ki}^2 and $\sigma_{MM_i}^2$ are, respectively, known as the single and multi-model variances.

D. Multi-model ensemble performance as a function of single-model diversity

In this study, we tested whether the performance of the multi-model ensemble was related to the diversity of the constituent single models. The results showed a negative correlation (correlation coefficient = -0.66) between the diversity of a single model and the multi-model performance improvement over the single model. Figure S1 plots the relationship of the diversity and improvement statistics pertaining to post-intervention predictions tabulated in Table 3 in the main text.

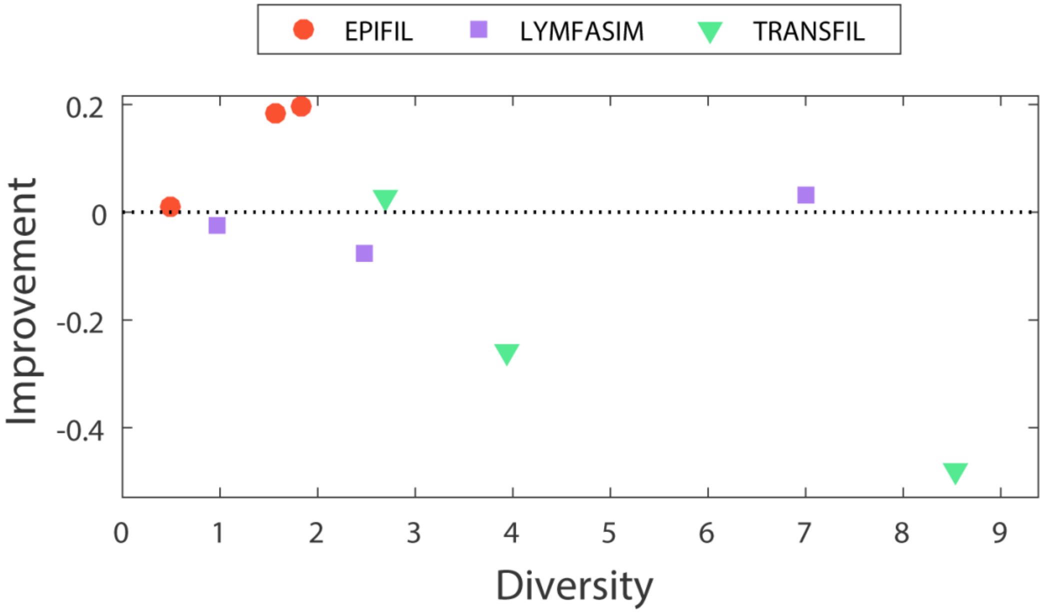

Figure S1 - Improvement in the performance of the MM ensemble over the single or individual (SM) ensembles in relation to diversity of the single models. There is a negative correlation (correlation coefficient = -0.66) between the diversity of the members of the single models and the improvement of the MM ensemble over the single models for forecasting and capturing the follow-up LF survey data.

E. Results of ensemble construction with bias correction

To account for systematic errors caused by model-specific uncertainties, such as variable model structures and parameter assumptions, a bias correction step can be performed on the single model ensembles before combining them to generate the multi-model ensemble. As an extension of the work presented in the main text, a linear transformation of the single model predictions was performed by fitting the coefficients a_k and b_k by least squares, and applying the correction as follows[23]:

$$x_{kni}^{c} = a_k + b_k x_{kni}$$

where x_{kni}^{c} is the bias-corrected prediction given by the ith age group of the member *n* of model *k*. In this exercise, all model weighting was carried out based on the bias-corrected model predictions, x_{kni}^{c}, which has been demonstrated elsewhere to significantly improve the predictive performance of a multi-model ensemble[23, 24].

The performance of the bias-corrected multi-model ensemble during the training period was slightly poorer when compared to the performance of the multi-model ensemble which has not been bias-corrected. Table S5 shows the comparison of evaluation statistics for the two scenarios with regard to the ability to fit to baseline mf age prevalence data. The assigned model weights were not significantly impacted by the use of bias correction (Table S6). In addition to negatively impacting the performance of the multi-model ensemble during the training period, the correction procedure contributed to a loss in prediction skill and diversity of the multi-model ensemble with regard to the post-intervention (validation) period. Figure S2 shows the single model and multi-model ensemble predictions with respect to the follow-up survey data available for each site. Note the poor prediction skill especially of the multi-model ensemble for Nanaha

(Figure S2B). Table S7 highlights where the bias-corrected ensemble was outperformed by the uncorrected ensemble.

Table S5. Multi-model ensemble performance in fitting the pre-intervention mf prevalence (training data). Evaluation statistics are given for two scenarios for comparison: 1) when a linear bias correction is performed on the single model predictions prior to generating the ensemble, and 2) when no bias correction is performed. Bolded values indicate which scenario performed better for the corresponding site and evaluation statistic.

Site	*ReRMSE*		*Diversity*	
	With BC	Without BC	With BC	Without BC
Malindi	0.507	**0.476**	2.707	**3.083**
Nanaha	1.380	**1.037**	6.729	**8.911**
Pondicherry	0.735	**0.685**	**2.580**	2.411

Table S6. Model weights given to each single model in generating the multi-model ensemble. Model weights are given for two scenarios for comparison: 1) when a linear bias correction is performed on the single model predictions prior to generating the ensemble, and 2) when no bias correction is performed.

Site	Model	Weight	
		With BC	Without BC
Malindi	EPIFIL	0.617	0.600
	LYMFASIM	0.279	0.287
	TRANSFIL	0.105	0.113
Nanaha	EPIFIL	0.349	0.318
	LYMFASIM	0.403	0.413
	TRANSFIL	0.248	0.269
Pondicherry	EPIFIL	0.141	0.171
	LYMFASIM	0.758	0.751
	TRANSFIL	0.101	0.077

Table S7. Multi-model ensemble performance in predicting post-intervention mf prevalence (validation data). Evaluation statistics are given for two scenarios for comparison: 1) when a linear bias correction is performed on the single model predictions prior to generating the ensemble, and 2) when no bias correction is performed. Bolded values indicate which scenario performed better for the corresponding site and evaluation statistic.

Site	*ReRMSE*		*Diversity*		*Average Improvement*	
	With BC	Without BC	With BC	Without BC	With BC	Without BC
Malindi	**0.669**	0.888	2.091	**2.105**	**0.214**	-0.107
Nanaha	1.480	**1.068**	11.283	**13.645**	-0.490	**-0.084**
Pondicherry	0.960	**0.927**	1.774	**1.892**	-0.017	**0.062**

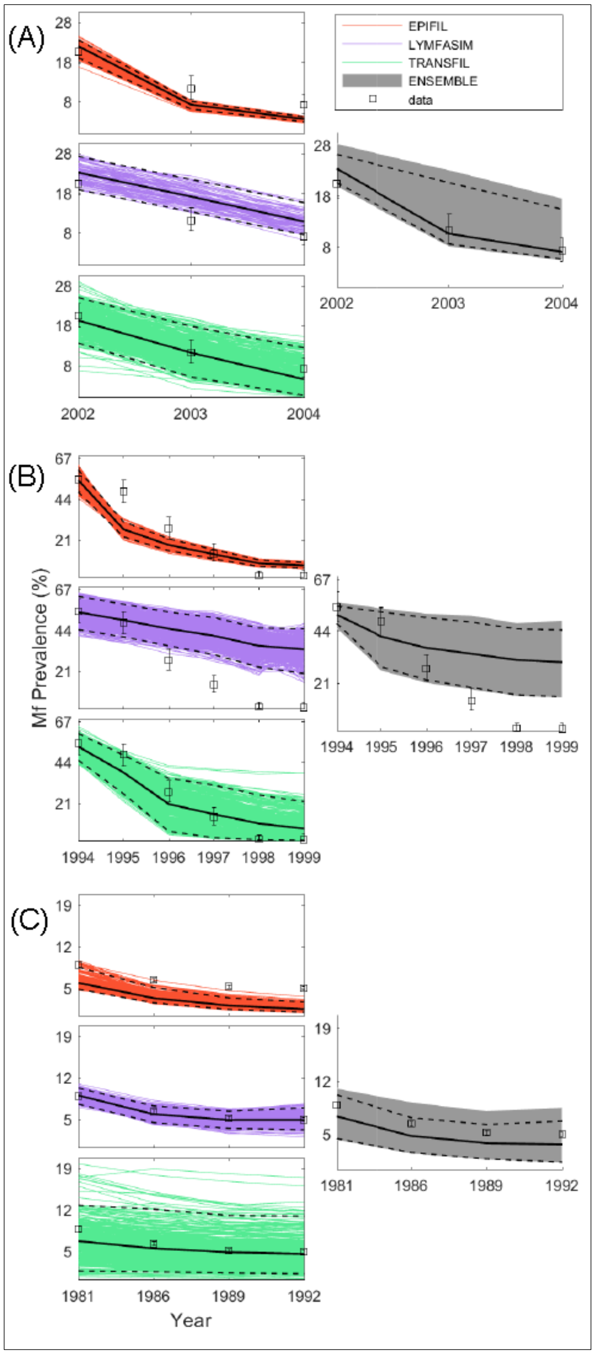

Figure S2. Intervention simulations by the single and bias-corrected multi-model ensembles for Malindi, Kenya (A), Nanaha, PNG (B), and Pondicherry, India (C). The observed age-stratified prevalence data are shown as open black *squares* with 95%-CI bands. The mean predictions are shown by *solid black lines*, and the 2.5% and 97.5% predictions are illustrated by *dashed black lines*. This figure mirrors Figure 3 in the main text showing results which have not been bias corrected.

References

1. Scott AL: **Lymphatic-dwelling Filariae.** In *Lymphatic Filariasis.* Edited by Nutman TB. London: Imperial College Press; 2000:5-39.

2. Subramanian S, Stolk W, Ramaiah K, Plaisier A, Krishnamoorthy K, Van Oortmarssen G, Amalraj DD, Habbema J, Das P: **The dynamics of Wuchereria bancrofti infection: a model-based analysis of longitudinal data from Pondicherry, India.** Parasitology 2004, **128**(05):467-482.

3. Vanamail P, Subramanian S, Das PK, Pani SP, Rajagopalan PK: **Estimation of fecundic life span of Wuchereria bancrofti from longitudinal study of human infection in an endemic area of Pondicherry (south India).** Indian J Med Res 1990, **91**(July):293-297.

4. Evans DB, Gelband H, Vlassoff C: **Social and economic factors and the control of lymphatic filariasis: A review.** Acta Trop 1993, **53**(1):1-26.

5. Ottesen EA, Ramachandran CP: **Lymphatic Filariasis Infection and Disease - Control Strategies.** Parasitol Today 1995, **11**(4):129-131.

6. Vanamail P, Ramaiah KD, Pani SP, Das PK, Grenfell BT, Bundy DAP: **Estimation of the fecund life span of Wuchereria bancrofti in an endemic area.** Trans R Soc Trop Med Hyg 1996, **90**(2):119-121.

7. Gambhir M, Michael E: **Complex ecological dynamics and eradicability of the vector borne macroparasitic disease, lymphatic filariasis.** PLoS One 2008, **3**(8):e2874.

8. Gambhir M, Bockarie M, Tisch D, Kazura J, Remais J, Spear R, Michael E: **Geographic and ecologic heterogeneity in elimination thresholds for the major vector-borne helminthic disease, lymphatic filariasis.** BMC Biol 2010, **8**:22.

9. Singh BK, Bockarie MJ, Gambhir M, Siba PM, Tisch DJ, Kazura J, Michael E: **Sequential modeling of the effects of mass drug treatments on Anopheline-mediated lymphatic filariasis infection in Papua New Guinea.** PLoS One 2013, **8**(6):e67004.

10. Hairston NG, de Meillon B: **On the inefficiency of transmission of Wuchereria bancrofti from mosquito to human host.** Bull World Health Organ 1968, **38**(6):935-941.

11. Subramanian S, Krishnamoorthy K, Ramaiah KD, Habbema JDF, Das PK, Plaisier AP: **The relationship between microfilarial load in the human host and uptake and development of *Wuchereria bancrofti* microfilariae by *Culex quinquefasciatus*: a study under natural conditions.** Parasitology 1998, **116**(03):243-255.

12. Subramanian S, Pani SP, Das PK, Rajagopalan PK: **Bancroftian Filariasis in Pondicherry, South India: 2. Epidemiological Evaluation of the Effect of Vector Control.** Epidemiol Infect 1989, **103**(3):693-702.

13. Das PK, Manoharan A, Subramanian S, Ramaiah KD, Pani SP, Rajavel AR, Rajagopalan PK: **Bancroftian Filariasis in Pondicherry, South India: Epidemiological Impact of Recovery of the Vector Population.** Epidemiol Infect 1992, **108**(3):483-493.

14. Ho BC, Ewert A: **Experimental transmission of filarial larvae in relation to feeding behaviour of the mosquito vectors.** Trans R Soc Trop Med Hyg 1967, **61**(5):663-666.

15. Norman RA, Chan MS, Srividya A, Pani SP, Ramaiah KD, Vanamail P, Michael E, Das PK, Bundy DA: **EPIFIL: the development of an age-structured model for describing the transmission dynamics and control of lymphatic filariasis.** Epidemiol Infect 2000, **124**(3):529-41.

16. May RM: **Togetherness among Schistosomes: its effects on the dynamics of the infection.** Math Biosci 1977, **35**(3–4):301-343.

17. Rajagopalan PK: **Population dynamics of Culex pipiens fatigans, the filariasis vector, in Pondicherry - influence of climate and environment.** Proc Ind Nat Science Acad 1980, **B46**:745-752.

18. Subramanian S, Manoharan A, Ramaiah KD, Das PK: **Rates of Acquisition and Loss of Wuchereria Bancrofti Infection in Culex Quinquefasciatus.** Am J Trop Med Hyg 1994, **51**(2):244-249.

19. Michael E: **The epidemiology of filariasis control.** In *World Class Parasites: the Filaria.* Edited by Klei TR, Rajan TV. Boston: Kluwer Academic Publishers; 2002:60-81.

20. Michael E, Malecela-Lazaro MN, Simonsen PE, Pedersen EM, Barker G, Kumar A, Kazura JW: **Mathematical modelling and the control of lymphatic filariasis.** Lancet Infect Dis 2004, **4**(4):223-34.

21. Duerr HP, Dietz K, Eichner M: **Determinants of the eradicability of filarial infections: a conceptual approach.** Trends Parasitol 2005, **21**(2):88-96.

22. Johnson C, Swinbank R: **Medium-range multimodel ensemble combination and calibration.** Q J R Meteorol Soc 2009, **135**(640):777-794.

23. Watanabe S, Kanae S, Seto S, Yeh PJ, Hirabayashi Y, Oki T: **Intercomparison of bias-correction methods for monthly temperature and precipitation simulated by multiple climate models.** Journal of Geophysical Research 2012, **117**(D23).

24. Teutschbein C, Seibert J: **Bias correction of regional climate model simulations for hydrological climate-change impact studies: Review and evaluation of different methods.** Journal of Hydrology 2012, **456**:12-29.
